# Towards Enhanced Electrospinning of Alginate—Can Recent Strategies Overcome Limitations? A Review

**DOI:** 10.3390/polym17162255

**Published:** 2025-08-20

**Authors:** Paulina Wróbel, Julia Zwolińska, Daniel Szopa, Anna Witek-Krowiak

**Affiliations:** Department of Engineering and Technology of Chemical Processes, Faculty of Chemistry, Wroclaw University of Science and Technology, Gdanska 7/9, 50-344 Wroclaw, Poland; julia.zwolinska@pwr.edu.pl (J.Z.); daniel.szopa@pwr.edu.pl (D.S.)

**Keywords:** electrospinning, alginate, nanofibers, biopolymers, functionalization

## Abstract

Electrospun alginate nanofibers are emerging as versatile materials for biomedical, environmental, and packaging applications due to their biocompatibility, biodegradability, and functional tunability. However, the direct electrospinning of alginate remains a significant challenge, mainly due to its polyelectrolytic nature, rigid chain structure, and limited chain entanglement. This review provides a comprehensive analysis of recent strategies developed to overcome these limitations, including polymer blending, chemical modification, the addition of surfactants, multi-fluid techniques, and process optimization. We systematically discuss the integration of nanofibers with functional agents such as microorganisms, bioactive compounds, plant extracts, and nanoparticles, highlighting their potential in wound healing, active packaging, bioremediation, and controlled release systems. This review also examines the scalability of alginate electrospinning, summarizing recent patents, industrial solutions, and challenges related to the standardization of the process. Key knowledge gaps are identified, including the need for long-term stability studies, structure–function correlations, green processing approaches, and expansion into novel application domains beyond healthcare. Addressing these research directions will be crucial to unlocking the full potential of alginate nanofibers as sustainable, high-performance materials for industrial use.

## 1. Introduction

**Figure 1:**
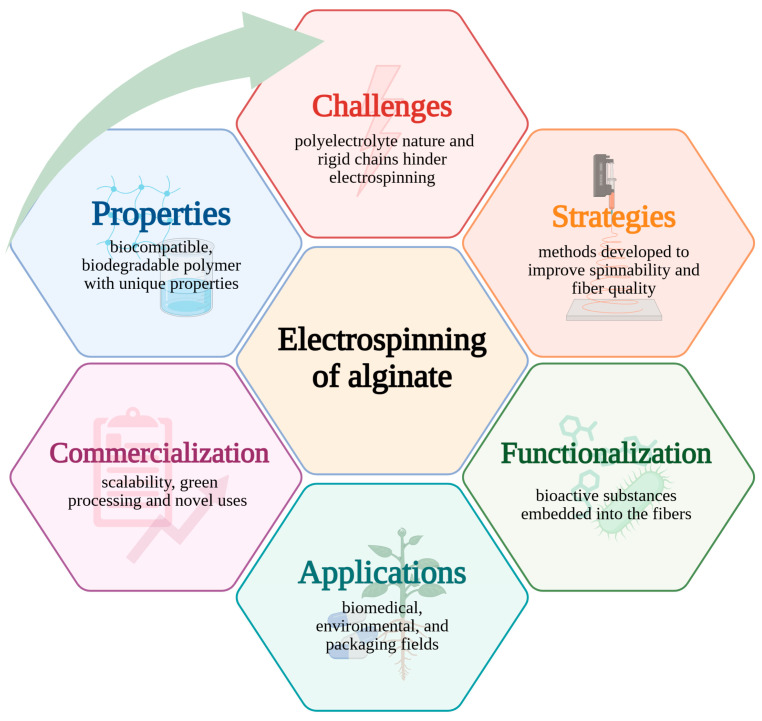
Overall concept of review. Created in Biorender.com, Zwolińska, J. (2025) https://BioRender.com/4280yjf, accessed on 14 August 2025.

Polymers, especially biopolymers, are increasingly explored due to their adaptability, biodegradability, and biocompatibility, which render them an eco-friendly alternative to synthetic materials derived from petroleum resources [1]. They may appear in diverse forms, including microbeads, films, or fibers, which allows their adaptation to specific applications. Electrospinning is a versatile technique for producing polymeric fibers with nanoscale diameters using a high-voltage electric field. This approach draws attention due to its capacity to optimize the fiber structure, providing specific mechanical and functional features [2]. These fibers have been identified by their increased surface/volume ratio, their superior permeability, and the ability to modify their structure at both the nanoscale and microscale. Due to these unique characteristics, electrospun fibers have found novel applications in the medical field [3], environmental protection [4], packaging [5], and agriculture [6]. The structure and the main points of this review are shown in Figure 1.

**Figure 2:**
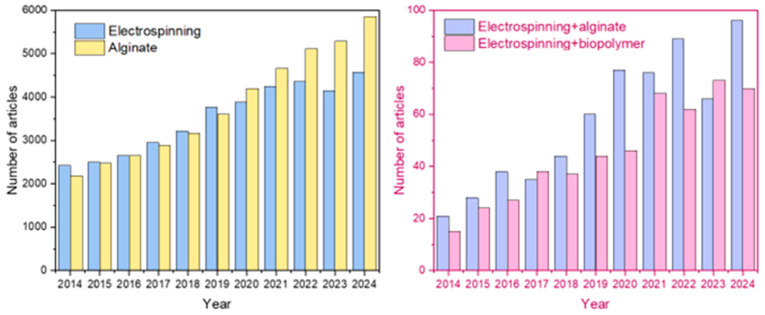
Web of Science database search result (accessed 17 February 2025), keywords “electrospinning”, “alginate” “electrospinning AND alginate”, and “electrospinning AND biopolymer” searched in abstract, title, and keywords.

The growing interest in the electrospinning of biopolymers, particularly alginate, is reflected in the number of publications indexed in Web of Science, which highlights the dynamic development of this research field and its significance for biomaterials engineering applications (Figure 2). Electrospinning of biopolymers such as sodium alginate (SA) encounters numerous challenges due to their polyelectrolytic nature and repulsive electrostatic interactions. These factors, along with bidirectional hydrogen bonding, create rigid, elongated chains in water, preventing stable fiber formation [7]. To enhance the spinnability of biopolymers, strategies such as blending them with other polymers, adding surfactants, and employing advanced electrospinning techniques, including multi-fluid methods, are utilized [8,9]. Researchers are also exploring green solvents and optimizing the electrospinning process to improve the efficiency of nanostructure production.

**Figure 3:**
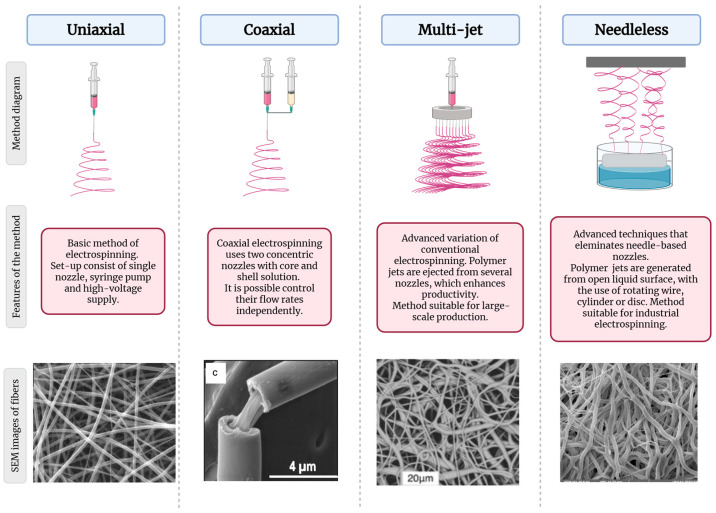
Variations overview of electrospinning models of polymeric fibers. Reproduced from ref. [17] copyright©2024; ref. [18] copyright©2020; ref. [19] copyright©2017; ref. [20] copyright © 2025 Elsevier. Created in Biorender.com. Witek-Krowiak, A. (2025) https://BioRender.com/74zd2vk, accessed on 14 August 2025.

Despite numerous studies on alginate electrospinning, several technological and functional challenges remain unresolved. Literature reviews to date have primarily focused on the biomedical aspects of electrospinning [9,10,11]. They analyze its applications in bioscaffolds for tissue engineering, in drug carriers, and in medical packaging. It has been shown that alginate nanofibers can be functionalized with antimicrobial additives such as nanoparticles (NPs) or antibiotics, which makes them promising materials for wound dressings [12,13]. Another paper analyzed the potential of electrospinning fibers from plant-, algal-, microbial-, and animal-derived biopolymers, with particular emphasis on their structure, properties, and applications. Process parameters, solvent effects, and mechanical properties of nanofibers were discussed, along with their potential applications in filtration, medicine, sensors, energy storage, and catalysis [14]. Electrospinning alginate with bacteria facilitates the formation of biocompatible nanofibers that promote the survival of microorganisms due to the natural properties of alginate when it is used as a component of bacterial biofilms [15]. Such structures are finding applications in probiotic delivery, tissue engineering, and controlled release of bacteria in medicine and biotechnology. Alginate shows potential in agriculture due to its ability to release active ingredients in a controlled manner, thereby improving the water retention in soil and promoting plant growth while reducing nutrient losses. Its biodegradable properties make it suitable for use as an environmentally friendly alternative to synthetic agrochemical carriers, which allows for reducing negative environmental impacts [16]. The different electrospinning models for polymeric fibers are presented in Figure 3.

The continuous increase in scientific output, particularly over the last decade, reflects not only the interdisciplinary potential of alginate-based nanofibers but also the technological advancements in process control, scalability, and material functionalization. This review aims to address significant research gaps and emerging opportunities within the field of alginate electrospinning. Despite considerable progress over the past decade, a comprehensive analysis of advanced electrospinning methods, optimal additives for fiber formation, and sustainable electrospinning practices remains lacking. In particular, critical factors such as the origin of alginate, its molecular weight, and the mannuronic/guluronic acid (M/G) ratio remain insufficiently explored in the context of fiber properties and practical applications. Furthermore, previous reviews have primarily focused on biomedical applications, with limited attention being given to promising interdisciplinary fields such as environmental protection, agriculture, and biodegradable food packaging. By integrating recent technological developments with a broader spectrum of potential applications, this review provides clear guidance on key areas that require further investigation, thus supporting future advancements and commercialization of alginate electrospinning technologies.

## 2. Alginate: Structure, Properties, and Potential in Electrospinning Applications

Alginate is a natural anionic polysaccharide consisting of a linear copolymer of β-D-mannuronic acid (M) and α-L-guluronic acid (G), which is linked by β-(1→4) glycosidic bonds. The distribution of blocks in alginate depends on various factors, such as its origin and harvesting time, which also affect its M/G ratio and molecular weight, and thus its physicochemical properties [21]. The primary source of alginate is brown algae, such as *Laminaria hyperborea*, *Ascophyllum nodosum*, or *Macrocystis pyrifera*, from which up to 40% of the dry matter can be obtained as alginate [22]. An alternative source is bacteria, such as *Azotobacter vinelandii* and *Pseudomonas aeruginosa*, but their use in industrial production remains limited [23]. Alginate is typically extracted from brown algae through a sequential process of acid and alkaline treatments followed by purification. Bacterial sources exist but are rarely used industrially [24]. Commercially available alginates exhibit molar masses ranging from 34 to 500 kDa, and their viscosity in 1% solutions ranges from 10 to 1000 mPa⸱s [24,25].

**Figure 4:**
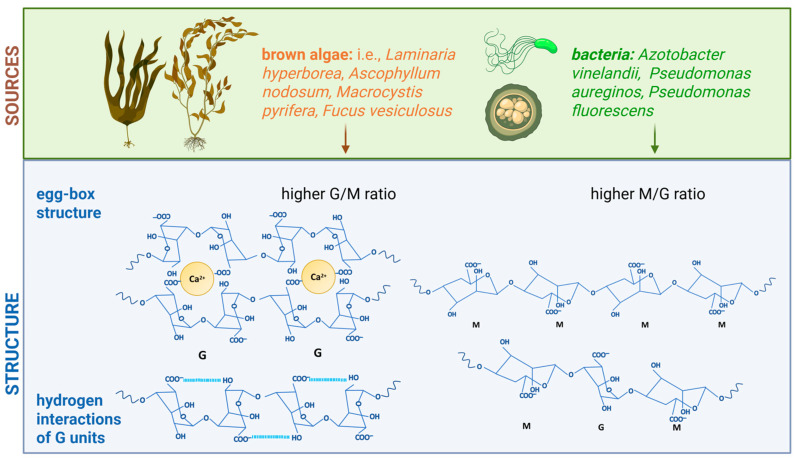
Alginate sources and molecular structure. Created in Biorender.com. Witek-Krowiak, A. (2025) https://BioRender.com/du2gwcm, accessed on 14 August 2025.

Alginates exhibit unique properties that make them an attractive material for various applications. Their ability to form hydrogels in the presence of calcium ions is attributed to the “egg-box” mechanism, where G-blocks of the polymer interact with Ca^2+^ ions, creating stable gel networks (Figure 4). Being biocompatible, biodegradable, and non-toxic, alginates are widely utilized in wound dressings, drug delivery systems, and tissue engineering [22]. Due to its water retention, low oxygen permeability, and adsorption capacity, alginate is also applied in packaging, filtration, and water treatment [26]. Alginate also presents a strong adsorption capacity, which makes it suitable for water purification, membrane production, and other environmental applications [13]. However, its mechanical strength, particularly in its hydrated state, remains limited, which poses a challenge for many engineering implementations [21].

Alginates from algal and bacterial sources exhibit distinct structural and chemical properties, influenced by their monomer composition and environmental factors, which significantly impact their functionalities and applications. Alginates derived from algae are characterized by a high content of guluronic blocks, and gel formation via Ca^2+^–G-block interactions enhances their structural integrity in biomedical applications [27]. The relationship between chain rigidity and alginate concentration is observed at high alginate concentrations in Na^+^ solutions, resulting from chain association [28]. Bacterial alginates contain a higher proportion of mannuronic blocks, which provide immunogenic properties and can enhance cytokine synthesis [29]. The dominance of M blocks can result in the formation of more flexible and brittle structures [27]. In addition to these intrinsic properties, alginate has garnered considerable attention in recent years due to its role in fabricating electrospun three-dimensional structures for biomedical applications.

Electrospinning produces nanofibers with a high surface area and an extracellular matrix-like architecture, which makes them ideal for use in biomedical applications. This technique has been increasingly adapted to obtain advanced 3D constructs, where both mechanical and biological properties are of key importance. Standard electrospun 3D designs include core-shell, bilayer, and multilayer composites tailored from natural (e.g., alginate, gelatin) and synthetic (e.g., polycaprolactone (PCL), PLA) polymers [30]. An example is the bilayer dressing, in which the outer layer, composed of nanofibrillar PCL loaded with an antibacterial drug, simulates the epidermal barrier. The inner layer, in the form of a hydrogel composed of alginate and gelatin enriched with growth factors, replicates the skin structure [31]. Core-shell systems offer controlled release by enclosing actives in the core, while the shell limits burst diffusion [32]. Applications of this approach include wound dressings and cartilage tissue regeneration systems, where nanofibers with a core-shell structure can stimulate stem cell differentiation [33]. However, producing advanced 3D layered structures requires detailed control of the parameters of electrospinning solutions. Precise tuning of viscosity, surface tension, and conductivity is essential for uniform fiber formation in 3D structures [31,34].

Coaxial electrospinning is commonly used for core-shell fibers with bioactive cores and diffusion-controlling shells (e.g., PCL) [32,33]. This technique uses a coaxial needle setup in which two polymer solutions are electrospun concurrently through concentric capillaries. One solution forms the fiber’s core, while the other creates an outer shell. The high-voltage electrostatic field draws both solutions together into a single compound jet, yielding continuous fibers with a distinct core-shell morphology that enables the segregation of functionalities between layers, such as a drug-loaded core and a protective shell. This allows for a prolonged, controlled release, which is imperative in medical applications such as wound healing or cartilage tissue regeneration. Further innovative methods, such as random electrospinning, allow the production of nanofibers with precisely arranged internal layers (nanolamines) [35,36]. In this process, advanced spinneret designs or dynamic multi-fluid feeding techniques are employed to introduce multiple polymer streams into a single spinning process, which results in a single jet that contains distinct polymer phases. As this jet elongates and solidifies, the different polymer phases self-arrange into separate nanometric layers within each fiber, effectively producing nanolaminated fibers. This internal layering enables each fiber to carry multiple functional components in discrete layers, allowing for complex, controlled release profiles or enhanced mechanical performance. Another development aspect is integrating the electrospinning technique with 3D printing technology. This hybrid approach enables the production of composite hybrid structures with complex, multilayered geometries that more closely mimic the natural tissue architecture. This hybrid method involves sequentially combining layers of electrospun fibers with layers of 3D-printed biomaterial, typically by depositing nanofiber mats onto a printed scaffold or alternating fiber and printed layers during fabrication. This combined approach enables the fabrication of scaffolds with a hierarchical, multi-scale architecture. The 3D-printed components create a defined macroscopic framework, while electrospun fiber layers provide nanoscale porosity and bioactive interfaces; together they form a multi-layered structure that closely mimics native tissue architecture. For instance, a composite skin dressing composed of 3D-printed alginate–gelatin and electrospun PCL provides mechanical support and sustained bioactive release [31,32]. Extended 3D systems also require chemical modification of polymers to improve their biological compatibility and mechanical stability. For example, sulfatization of alginate enables the weakening of strong hydrogen bonds between polymer chains, thereby enhancing electrospinning while also stimulating differentiation processes in cartilage tissue [33,37]. Thanks to such modifications, it is possible to obtain structures with optimized porosity and mechanical properties that are ideal for tissue engineering applications.

The electrospinning of advanced 3D layered structures is based on the synergy between traditional electrospinning techniques, 3D printing, and innovative methods such as random electrospinning. The key element is the appropriate selection and modification of polymer solutions, both synthetic (PCL, PLA) and natural (alginate, gelatin, chitosan), which enable the acquisition of materials with ideal mechanical properties, high porosity, and controlled drug release capabilities. Despite existing challenges, such as controlling solution parameters, spinnability, or mechanical stability, the progress in this field and its integration with other production technologies provide a solid foundation for further innovations. This rapidly evolving field shows strong potential for broad application in tissue engineering, drug delivery systems, and environmental applications [31,32,33,34,37].

## 3. Electrospinning of Alginate-Based Fibers

SA exhibits low electrospinnability, primarily due to its unique physicochemical properties. The rigidity of the alginate chain arises from the presence of G units, which, through biaxial bonding and stabilizing hydrogen interactions in aqueous solutions (Figure 4), impart a structure comparable to cellulose. Therefore, introducing a highly polar solvent or blending alginate with a more flexible polymer, such as polyethylene oxide (PEO), which interacts favorably with alginate, particularly those rich in M units, is essential for improving the resulting material’s flexibility and promoting effective chain entanglement [8,38]. The electrical conductivity of alginate is relatively high because its polyanionic carboxylate groups dissociate in water into mobile ions. These ions serve as effective charge carriers, even though alginate lacks conjugated (electron-conductive) structures. The presence of negatively charged groups along the polymer backbone induces electrostatic repulsion, which forces the chains into an extended conformation [8]. This phenomenon hinders the stable formation of fibers, resulting in discontinuous or irregular structures. The addition of salts can enhance spinnability; however, in the case of divalent ions, it may also promote gelation [39]. This occurs because divalent cations, such as Ca^2+^, can bridge alginate chains by binding to guluronic acid residues, which leads to premature ionic crosslinking and gel formation in the spinning solution.

### 3.1. Influence of Molecular Weight and M/G Ratio on Alginate Electrospinning

The molecular weight of sodium alginate (SA) strongly affects the electrospinning efficiency by modulating solution viscosity and chain entanglement. Higher molecular weights generally improve the mechanical strength but may impair spinnability, which has prompted the frequent use of alginate in blends with other polymers. This is because longer polymer chains increase the viscosity of solutions and number of intermolecular interactions, making the jet harder to stretch and stabilize during electrospinning. As a result, fiber formation becomes less uniform, and defects such as beads or jet breakage may occur. Pure alginate is rarely electrospinnable due to its limited chain flexibility, which is influenced by its uronic acid composition and solvent-induced repulsion between charged chains [40] (Table 1).

The higher the stiffness, the lower the possibility of forming a dense network. Stiff polymer chains exhibit low conformational flexibility due to intramolecular interactions such as hydrogen bonding or electrostatic repulsion. This restricts their ability to form transient entanglements with neighboring chains, reducing the chain overlap needed for network formation and stable fiber production. In aqueous solutions, the polyelectrolytic nature of alginate leads to interchain repulsion [37], which reduces entanglement and fiber continuity, potentially causing needle clogging during spinning. Molecular weights below 100 kDa exhibit high electrospinning instability due to the formation of short polymer chains, which results in fibers that display insufficient elasticity and favor the formation of polymer droplets rather than continuous nanofibers [40]. Studies have not definitively established the effect of the molecular weight of pure alginate on the electrospinning process. The rheological properties of solutions containing alginate and another polymer are mainly analyzed. It has been determined that the optimal molecular weight for an electrospinning solution ranges from 200 kDa to 500 kDa, which minimizes defects in the produced fibers [37,40].

For very-high-molecular-weight alginates, partial enzymatic hydrolysis is one strategy to improve their electrospinning properties by reducing the molecular weight to an optimal range [25,50]. Alternatively, diluting the alginate solution effectively lowers its viscosity, thus permitting electrospinning even with high-molecular-weight alginates [45,59,66]. The molecular weight of alginate also impacts the mechanical properties of the electrospun fibers. Alginates with a molecular weight above 250 kDa tend to produce fibers with higher mechanical strength, which is critical for biomedical applications, especially in tissue engineering [50,55,63]. As for alginates below 150 kDa, they often yield fibers with reduced mechanical strength, which potentially limits their use in applications that require high durability [57,58]. Additionally, studies on alginate oxidation have shown that oxidation can alter the molecular weight of alginate. Oxidation significantly reduces the molecular weight of alginate, changing its rheological and electrospinning properties. Higher degrees of oxidation correlate with increased degradation and reduced fiber strength. This indicates that oxidation reduces the molecular weight and alters the fiber-forming properties of alginate, as well as its physical and rheological properties, which are crucial factors when adjusting the composition of electrospinning solutions. Different types of alginates derived from various algae exhibit distinct structural features, including distinct molecular weights and block compositions, which in turn affects their ability to promote bone–cell adhesion. For instance, alginates with strong polyelectrolytic behavior (isolated from *Macrocystis piryfera*) significantly enhanced osteoblast adhesion, whereas, concerning skin cells, the differences among alginates ng skin cells (fibroblasts and keratinocytes) were less notable [50]. Most research emphasizes the biological effects of alginate rather than its spinning behavior. Future work should prioritize structural modifications to enhance its spinnability and process control.

The ratio of mannuronic to guluronic acid blocks determines the fiber elasticity and electrospinning performance, with intermediate values offering a favorable balance between flexibility and crosslinking. Data from the literature indicate that the M/G ratio in studied alginates ranges from 0.42 [42,44] to 2.65 [73], revealing a broad scope for modifying their properties. Ratios close to 1.56 [8,25,67,69,70,71,74] are among the most common in electrospinning systems, which indicates that alginate exhibits optimal electrospinning behavior in this range.

M/G ratios below 1, such as 0.42 [42,44], 0.43 [34,46], and 0.64 [51], indicate a dominance of guluronic acid. This composition enhances the stability of ionic crosslinking but simultaneously reduces the elasticity and processability of the material during electrospinning. This is because guluronic acid blocks form more rigid and linear chains, which favor strong ionic interactions with calcium ions but reduce the flexibility and entanglement of the polymer chains in solution. As a result, the formation of continuous nanofibers is hindered, leading to unstable jets or bead formation during the electrospinning process. Such alginate may require modification by blending with other polymers to improve its rheological properties. However, higher M/G ratios (e.g., 1.61 [43], 2.33 [72], or 2.65 [73]) reflect a predominance of mannuronic acid, which enhances the fiber’s elasticity and extensibility. An excessive amount of M segments can lower the capacity for ionic crosslinking because mannuronic acid residues adopt a more flexible, linear conformation that lacks the specific spatial arrangement of carboxyl and hydroxyl groups, which is needed to cooperatively chelate calcium ions. Additional chemical crosslinking or hybrid polymer systems are needed to complete the electrospinning process in this scenario. Regarding electrospinning, an M/G ratio in the range of 1.25–1.56 [52,53,54,56,58,62,64] seems most advantageous, as it balances elasticity and mechanical stability. Alginates within this ratio range can be used in pure form or in blends with other polymers (e.g., PEO or polylactic acid (PLA)), as they enhance both the electrospinning properties and the ultimate strength of the resulting fibers.

### 3.2. Improving Alginate Spinnability and Fiber Functionality 

#### Blending Alginate with Synthetic Polymers

Among the synthetic polymers blended with alginate to improve its electrospinnability, PEO is most commonly used, followed by polyvinyl alcohol (PVA) (Table 2).

### 3.3. Improving Alginate Spinnability and Fiber Functionality

#### 3.3.1. Blending Alginate with Synthetic Polymers

Among the synthetic polymers that are blended with alginate to improve its electrospinnability, PEO is the most commonly used, followed by polyvinyl alcohol (PVA) (Table 2). PEO and PVA improve alginate’s electrospinnability by reducing interchain repulsions and promoting hydrogen bonding, which enhance chain flexibility. They also reduce the viscosity and conductivity of alginate solutions, facilitating more stable fiber formation [82,83].

PEO is a hydrophilic polymer that is widely used due to its biocompatibility, biodegradability, and non-toxicity, with a molecular weight ranging from 100,000 to 7,000,000 g/mol [83]. PEO solutions exhibit viscosity values that depend on both their molecular weight and concentration. A lower molecular weight at high concentrations results in higher viscosity, whereas high-MW PEO at low concentrations shows reduced viscosity [84]. PEO is a suitable additive for alginate blends due to its high solubility, which results from the strong hydrogen bonding between the carboxyl groups of alginate and the ether groups of PEO. These hydrogen bonds increase the solution’s electrical conductivity and reduce its surface tension. Electrospinning SA with high-molecular-weight PEO (4 MDa), where the total polymer concentration is 3.5% and the PEO content is 10%, can yield nanofibers containing even 90% SA [7]. An electrospinning solution containing 2.5% SA (58.9 kDa) and 1.5% PEO (600 kDa) showed that, although using lower-molecular-weight PEO produces smooth, bead-free nanofibers, it substantially reduces the achievable alginate content in the resulting fibers [73]. PVA, similar to PEO, is a water-soluble, biocompatible, and biodegradable synthetic polymer and its molecular mass also affects fiber production [85]. Examination of different molecular weights of PVA, ranging from 67 to 168 kDa, revealed that higher molecular masses (146–186 kDa) yield more uniform fibers. The viscosity of PVA (<400 kDa) solutions increases significantly with higher polymer concentrations, ranging from 5 cP at 2.5% PVA to 82 cP at 10.5% PVA [86]. PVA forms stronger and more compact hydrogen bonds than PEO due to its high content of hydroxyl groups, which enhances the strength and stability of the resulting fiber. Additionally, PVA forms semi-crystalline structures, which increase the mechanical strength and stiffness of the fibers compared to the amorphous nature of PEO. This is because segments of PVA chains can arrange themselves in a more ordered fashion, forming crystalline regions stabilized by intermolecular hydrogen bonds. These domains act as reinforcing points within the fiber structure, limiting chain movement and enabling the material to better withstand mechanical stress. In contrast, the fully amorphous structure of PEO lacks such reinforcement, resulting in fibers that are more flexible but mechanically weaker. It is worth noting that the concentration of PVA also plays a crucial role in electrospinning, as only smooth fibers were produced at concentrations of 5–7 wt.%, whereas, at concentrations between 10 and 20 wt.%, the fiber uniformity decreased, which resulted in flat, non-uniform, and thicker fibers with several interconnected web-like structures [87]. To the best of our knowledge, the influence of the molecular mass of PVA on electrospinning with alginate has not been investigated; however, we expect its effects to be analogous to those observed with PEO. Blending a 2% SA solution with a 10% PVA solution (Mw 70,000–100,000 g/mol) at various volume ratios (0:10, 1:9, 2:8, and 3:7) resulted in beadless nanofibers; however, increasing the alginate content beyond a 4:6 ratio produced beaded fibers, likely due to the dominant polyelectrolyte nature of alginate [62]. Alginate’s strong polyelectrolyte character leads to electrostatic repulsion between its negatively charged chains in aqueous solution. This repulsion prevents the chains from coming close enough to physically entangle, a critical factor for maintaining a stable and continuous electrospinning jet. The polymer solution breaks up during spinning without adequate entanglement, forming beads instead of smooth nanofibers. Alginate is insoluble in non-aqueous solutions and PEO becomes soluble in ethanol above a temperature of 60 °C. This property enables the selective removal of PEO from alginate–PEO electrospun fibers by washing them with ethanol, which results in fibers composed solely of alginate [50].

Both PEO and PVA improve the electrospinning of alginate by reducing the viscosity of the solution and increasing chain entanglement. PEO allows for higher alginate content in the resulting fibers, while PVA offers greater mechanical strength due to stronger intermolecular interactions.

#### 3.3.2. Blending Alginate with Biopolymers

Scientists also examined the addition of other biopolymers, including chitosan, gelatin, pullulan, and collagen. Chitosan and alginate form a polyelectrolyte complex through ionic interactions between oppositely charged groups, improving the solution’s conductivity and reducing its surface tension. The mechanism involves electrostatic attraction between protonated amine groups of chitosan (-NH_3_^+^) and deprotonated carboxyl groups of alginate (-COO^−^), which form ionic pairs. The release of counterions increases the entropy, while the resulting ionic crosslinking stabilizes the polyelectrolyte complex and contributes to the formation of a network in the solution. This enhances Taylor cone stability and the strength of the fiber. PEO is often added to modulate conductivity and further support stable electrospinning [79]. Carboxymethyl chitosan, a water-soluble derivative, forms strong ionic and hydrogen bonds with alginate, enhancing the scaffold’s strength and thermal stability [32].

The possibility of electrospinning fibers without synthetic polymers was also investigated by using a solution containing SA and pullulan, a water-soluble polysaccharide whose aqueous solutions exhibit relatively lower viscosity compared to pure alginate solutions. Incorporating up to 1.6% of SA into a 10% pullulan solution for use in the free-surface electrospinning method resulted in bead-free fibers. Alginate’s positive effect stems from its ability to boost polymer chain entanglement and strengthen hydrogen bonding interactions with pullulan [56]. This ability is the result of the electrostatic repulsion between negatively charged carboxylate groups on alginate chains, which forces them into an extended, rod-like conformation in aqueous solution. This conformation increases the exposure and accessibility of functional groups. Pure pullulan requires high concentrations for fiber formation due to its low viscosity and weak chain entanglement. Adding alginate enhances the conductivity of the solution and facilitates fiber formation at lower pullulan concentrations. An electrospinning solution with a composition of 10% pullulan and 2% SA was successfully electrospun using an uniaxial process [88].

Gelatin is a widely used biopolymer with excellent electrospinnability, even without the addition of polymer additives. Its amphoteric nature, resulting from the presence of carboxyl and amino groups, allows for pH-dependent charge tuning, enabling compatibility with various polymers. This reversible protonation/deprotonation allows the polymer to switch between positive, neutral, or negative charges depending on the environment. Incorporating small amounts of alginate or chitosan can further enhance the uniformity and mechanical strength of the resulting fiber. Lowering the gelatin concentration by increasing the alginate and chitosan levels decreases the solution’s viscosity and reduces polymer chain entanglements, negatively impacting spinnability [89]. This occurs because gelatin provides flexible chains that easily entangle, while alginate and chitosan form more rigid structures with limited chain mobility, reducing the formation of the continuous polymer network required for stable fiber formation. Nanofibers composed solely of gelatin and SA aqueous solutions have been successfully produced using centrifugal spinning. This technique utilizes centrifugal forces instead of the electric fields employed in traditional electrospinning. The most optimal fibers were obtained by adding 10% SA to a 20% gelatin solution. Notably, continuous heating of the spinning system with hot air is required to maintain elevated temperatures, which prevent premature gelation of the solution [90].

Collagen is one of the most common biodegradable and biocompatible biopolymers used in tissue engineering applications. Collagen at an acidic pH carries positively charged amino groups, enabling the formation of a polyelectrolyte complex with the negatively charged carboxyl groups of alginate. Similar to chitosan, the resulting complex increases the solution’s viscosity and conductivity while simultaneously lowering its surface tension, which are conditions essential for a stable electrospinning process. Nanofiber formation with collagen and alginate solutions is possible through coaxial electrospinning. Coaxial electrospinning of collagen- and alginate-based solutions yields biocompatible fibers with excellent mechanical properties and high cell viability, which makes them suitable for wound-healing applications. Coaxial electrospinning is particularly advantageous in this context because it enables the simultaneous spinning of collagen and alginate into a core-shell fiber structure. This technique allows each polymer to retain its distinct functional role within the same fiber. In this study, collagen was used as a natural scaffold, while SA served as an absorber of excess wound fluids. The 3-(4, 5-dimethylthiazol-2-yl)-2, 5-diphenyltetrazolium bromide (MTT) assay and cell morphology analysis confirmed that the nanofibers are biocompatible and non-toxic [81]. A notable approach involved the development of an amphoteric composite sponge composed of electrospun sodium alginate nanofibers and chitosan, which enabled the simultaneous adsorption of both cationic and anionic dyes. The resulting material shows strong potential as an efficient adsorbent for complex water treatment applications [77].

Blending alginate with biopolymers provides a bio-based alternative to synthetic additives, offering enhanced biocompatibility, pH responsiveness, and mechanical flexibility, properties essential for biomedical and environmental applications.

#### 3.3.3. Chemical Modification of Alginate

To enhance the properties of alginate for improved electrospinnability and broader application suitability, researchers have explored various methods of modification. For example, SA can be oxidized and subsequently reacted with 1,4-phenylenediamine to form Alg-NH_2_. The modified alginate undergoes oxidative polymerization with aniline, forming Alg-g-PANI copolymers. This process introduces conjugated π–electron systems into the polymer structure, significantly enhancing its electrical conductivity. As a result, the alginate-based nanofibers acquire electroactive properties, which makes them suitable for applications such as electrically responsive scaffolds or biosensors that require signal transduction or stimulation. When blended with PVA, these materials can be electrospun into conductive nanofibrous scaffolds. These scaffolds exhibit favorable electroactivity and conductivity, which makes them suitable candidates for tissue engineering applications [71]. The conductive network facilitates charge transfer and generates localized electric fields, which influence transmembrane potentials and activate voltage-gated ion channels in cells.

A simple thermal treatment method involving heating alginate in an oven at 120 °C for 2 h can effectively improve its electrospinnability. This process reduces the molecular weight of alginate, shortens its polymer chain lengths, and consequently decreases the solution’s viscosity. Additionally, this technique is widely accessible and avoids the use of toxic chemicals [78]. Amidation of alginate via coupling with octylamine in the presence of (1-ethyl-3-(3-dimethyl-aminopropyl) carbodiimide hydrochloride (EDC)·HCl) is a way to synthesize octyl-grafted amphiphilic alginate derivative (AAD). A comparison of SA/PVA and AAD/PVA nanofibers revealed that the AAD/PVA solution exhibited better electrospinnability. Fibers containing pure AAD were not obtained, but the amidation reaction enabled the production of fibers with a higher alginate content [91]. This occurs because amidation with hydrophobic alkyl amines reduces the overall charge density and hydrophilicity of alginate, limiting electrostatic repulsion and improving the solution’s viscosity and chain entanglement. Modifying alginate is a method for enhancing its limited electrospinnability.

#### 3.3.4. Surfactants as Additives

Non-ionic surfactants, such as Triton X-100 and Pluronic F-127, are commonly used to lower the surface tension of electrospinning solutions, enabling the formation of smoother, bead-free fibers [73]. Their effectiveness stems from their ability to accumulate at the solution–air interface and lower surface tension, which facilitates the stable formation of jets during electrospinning. Their non-ionic nature also prevents significant interactions with alginate chains. Among the most frequently used surfactants are Triton X-100, Pluronic F-127, and Span 80, which are typically applied at concentrations of around 1 wt%. Pluronic F-127 is particularly well-suited for biomedical use due to its lower toxicity compared to Triton X-100. Increasing its concentration from 0.5% to 1% results in more uniform fibers [92]. Moreover, surfactants enable higher alginate loading in alginate/PEO blends (up to 80%) without increasing the conductivity, thereby enhancing the overall quality and stability of the fiber [93].

### 3.4. Optimization of Electrospinning Parameters

Key electrospinning parameters include applied voltage, solution flow rate, needle-to-collector distance, and environmental conditions such as temperature and humidity. Standard operational ranges include voltages between 15 and 30 kV, flow rates of 0.1–2 mL/h, and needle-to-collector distances that are typically centered around 15 cm (Table 2). Fiber formation begins once the applied voltage exceeds the critical threshold to enable the initiation of the jet and subsequent fiber elongation. Voltages outside the optimal range impair electrospinnability and disrupt the fiber’s morphology by altering its diameter. Excessive or insufficient voltage disrupts fiber formation and affects its diameter. Similarly, longer distances or higher flow rates can produce thinner fibers, but may also reduce the stability of the process [75]. The interaction between the voltage, flow rate, and working distance has a significant impact on the fiber morphology. For instance, shorter distances combined with a higher voltage enhance the electric field strength, allowing for the formation of thinner fibers. The fiber diameter decreases with an increasing flow rate up to an optimal point (e.g., 0.6 mL/h), beyond which droplet formation and beading may occur. According to Wang et al. [94], the optimal conditions for producing the smallest fiber diameter were identified as a distance of 15 cm and a flow rate of 0.5 mL/h. Larger distances weaken the electric field, reducing fiber stretching, whereas excessive flow rates lead to instability due to incomplete evaporation of the solution. Results of electrospinning process optimization with a solution of 4% alginate/PEO (7:3) and a 0.5% addition of Triton X-100 showed that both the feed rate and applied voltage have the most significant influence on the process. At a fixed working distance and voltage, increasing the flow rate promoted droplet formation, which indicates insufficient solvent evaporation and fiber instability. Similarly, a lower applied voltage, while keeping the working distance and feed rate constant, also resulted in droplet formation [95]. Optimizing the morphology of fiber requires a fine-tuned balance between the voltage, distance, and flow rate.

The molecular weight and solution concentration are critical factors that influence alginate’s electrospinnability, as they determine the extent of polymer chain entanglement. High-molecular-weight alginate exhibits exceptional mechanical properties and is appropriate for applications like wound dressings; however, its elevated viscosity and robust hydrogen bonding frequently impede electrospinning unless the polymer is blended or chemically altered [9,93]. Uniform, bead-free nanofibers are generally produced when the polymer concentration surpasses the critical entanglement concentration (Ce), which guarantees adequate chain overlap and viscoelastic cohesion for jet elongation. Nonetheless, the polyelectrolyte characteristics and viscosity of alginate at these concentrations may restrict its spinnability unless co-polymers or surfactants are included. In contrast, low-molecular-weight alginate or solutions with concentrations below Ce exhibit insufficient chain networking, which results in the electrospinning jet fragmenting into droplets or beads instead of producing continuous fibers [96].

### 3.5. Crosslinking Methods for Enhanced Fiber Stability

Crosslinking improves the mechanical strength and water resistance of alginate fibers, expanding their utility in biomedical and environmental applications. While ionic gelation is rapid, additional stabilization is often necessary. Ionic crosslinking, typically using 1–3% calcium chloride, rapidly gels alginate via interactions between carboxyl groups and Ca^2+^ ions, following the “egg-box” model. Optimal results are often achieved with a 2% solution applied for 5–30 min [50]. The main advantage of this method is its simplicity and low toxicity, which is particularly important in biomedical applications. However, in this method, the ionic bonds are relatively weak and can be exchanged with ions in the physiological environment, which leads to the gradual dissolution of the matrix.

Covalent crosslinking creates permanent bonds between alginate chains, offering greater structural durability. A widely used method employs EDC/NHS chemistry, where 0.5–2% (*w*/*v*) EDC is combined with NHS in a 1:1 molar ratio. The reaction is conducted at room temperature for from 30 min to 2 h, producing stable amide linkages and significantly improving the mechanical resistance and water stability of the resulting fiber [97]. The Schiff base reaction involves oxidizing alginate to introduce aldehyde groups, which react with protein amines (e.g., gelatin) to form reversible imine bonds that are responsive to the environmental pH. Oxidation is typically performed using 0.1–0.5 M potassium permanganate heated at ~25 °C for 1–4 h, which is followed by the addition of protein (0.1–0.5% *w*/*v*) and a reaction at pH 5.5–7.0 for an additional 1–4 h. This method produces composite scaffolds with pH-responsive behavior, although their efficiency strongly depends on tightly controlled conditions [43]. Glutaraldehyde rapidly forms covalent networks but is toxic, which limits its application in biomedical products despite its efficiency [83]. TFA acts as a solvent and proton donor, crosslinking alginate by converting carboxylate groups to carboxylic acids. It also affects alginate’s composition (M/G ratio) and dissolves PEO [98]. Comparative studies show that each crosslinker yields different fiber characteristics. CaCl_2_-crosslinked membranes exhibit the highest tensile strength, while TFA-crosslinked fibers offer the most significant specific surface area and best performance in acidic environments. In contrast, GA-crosslinked membranes maintain good morphology and stability in alkaline and marine conditions [58]. These differences indicate that crosslinking methods should be selected based on the intended application environment.

Photocrosslinking involves the chemical modification of alginate with methacrylic groups, typically via methacrylic anhydride or glycidyl methacrylate, followed by UV-induced curing. This process is performed using 2–4% alginate solutions with 0.05–0.1% photoinitiator that are exposed to UV light (~10 mW/cm^2^) for 5–10 min. The method enables precise control over the network architecture and yields highly stable covalent structures, though it requires pre-functionalization, which may alter alginate’s native properties [42].

Crosslinking strategies significantly enhance the performance of nanofibers. Tailoring the crosslinking method allows customization of the fiber’s stability, mechanical strength, and responsiveness for specific applications.

### 3.6. Green Processing Approaches and Long-Term Stability

#### 3.6.1. Green Processing Strategies for Alginate Electrospinning

Ongoing research focuses on the development of sustainable, low-toxicity methodologies for electrospinning alginate fibers. A prominent strategy involves substituting volatile organic solvents with deep eutectic solvents (DES) or other environmentally benign alternatives. DES are formulated from natural, biodegradable constituents such as choline chloride combined with urea or glycerol and are capable of dissolving biopolymers while exhibiting negligible vapor pressure; thereby, they reduce hazardous emissions and enable efficient solvent recovery [99]. Using DES in electrospinning has been shown to produce nanofibers while avoiding hazardous reagents, and this strategy is now being explored [100]. Another sustainable approach is enzyme-assisted crosslinking [101] and the use of bio-based crosslinkers. For example, alginate can be blended with gelatin/chitosan, followed by crosslinking with natural crosslinkers like genipin (a plant-derived agent), to produce water-insoluble fiber mats [102]. Such naturally derived crosslinking methods greatly reduce the cytotoxicity and environmental impact of the process, though they may require additional steps to be integrated into the spinning process. A recent advancement in the field demonstrates that, in contrast to the conventional immersion of freshly electrospun alginate fibers in CaCl_2_/ethanol to prevent water-induced dissolution, glycerol–water mixtures or pH-controlled aqueous Ca^2+^ solutions can achieve effective ethanol-free crosslinking [73].

Green processing innovations, encompassing solvent systems such as DES or water and crosslinking via enzymes or other benign agents, collectively enhance the sustainability and safety of alginate nanofiber fabrication by eliminating toxic reagents, thereby reducing the environmental footprint of the process while preserving fiber formation efficiency.

#### 3.6.2. Long-Term Stability and Degradation of Alginate Nanofibers

The biodegradability of alginate is a double-edged sword; it ensures environmental friendliness but can compromise the long-term stability of fibers. In soil and natural water, crosslinked alginate nanofibers tend to break down over weeks to months via ionic dissolution and microbial digestion. Soil burial studies on alginate-based materials report substantial weight loss (around 80% degradation in 1–2 months) under active microbial conditions [103]. Experiments show that Ca–alginate fiber mats remain intact in pure water or acidic (stomach-mimicking) solutions, yet swell and gradually disintegrate in phosphate-buffered saline (PBS) as the crosslinks are diluted [73]. Current research gaps include the lack of real-time degradation studies of alginate nanofibers across diverse environmental and physiological conditions, as most existing work addresses only short-term stability in laboratory simulants, with limited data existing on long-term breakdown in various environments.

## 4. Applications of Electrospun Alginate Fibers

Thanks to their large surface area, porosity, and biocompatibility, electrospun alginate fibers are utilized in medicine, packaging, and environmental technologies, extending far beyond traditional biomedical applications. Encapsulation within alginate nanofibers offers protection for bioactive compounds and microorganisms against environmental stressors such as heat, light, and oxidation [104]. Electrospinning enables efficient nanoencapsulation while preserving biological activity [105], offering potential applications in drug delivery, food packaging, and the stabilization of probiotics.

### 4.1. Microorganism-Loaded Alginate Fibers

Despite its significant potential, there is a limited number of studies available on the integration of microorganisms into electrospun fibers [106,107]. Due to the specific requirements of microorganisms, the use of non-toxic, biocompatible polymers with good solubility in water or mildly acidic solutions is essential for maintaining microbial viability [108]. The selected microorganisms must withstand high voltage, remain viable at low concentrations, and meet safety standards (e.g., GRAS status) to ensure both process compatibility and functionality of electrospun fibers [109,110,111]. When considering the application of microorganism-loaded fibers, effectiveness against pathogens (e.g., *S. aureus* in skin infections) is a key feature. From an economic perspective, using inexpensive culture substrates for microbial propagation is the most efficient approach, directly impacting the process’s profitability.

Table 3 presents an overview of the latest studies on integrating microorganisms into alginate electrospun fibers. The majority of available studies on the electrospinning of microorganisms focus on probiotic bacteria, which the WHO (World Health Organization) and FAO (Food and Agriculture Organization) define as “live microbes, when administered in adequate quantities, confer health benefits on the host organism” [112]. Due to their sensitivity to environmental conditions and low survival rate, it is essential to apply protective measures to enhance their viability, both in the intestinal environment and in more challenging conditions [113]. Microorganisms are most commonly incorporated into fibers composed of alginate blended with synthetic polymers. A polymer matrix of PVA–alginate containing *Lactobacillus paracasei* K2-199 was electrospun at 22 kV and achievied a high post-processing survival rate of 85.87% [114], which is notably higher than that achieved by lyophilization, a well-known microbial preservation method [115]. During electrospinning, the rapid removal of solvent minimizes the time for which cells are exposed to environmental stress. Additionally, conducting the process at room temperature makes it suitable for encapsulating sensitive microorganisms. In contrast, during lyophilization, bacteria are subjected to dehydration, shear stress, and temperature changes, which can result in a loss of viability [116]. Under simulated gastrointestinal conditions, immobilized bacteria showed 20% higher viability than free cells. These results confirm alginate’s potential as a nanoencapsulation material that protects probiotics during processing and gastrointestinal passage and supports their delivery at effective daily doses (10^6^–10^9^ CFU/g) [117].

Another example of using probiotic bacteria in alginate nanofibers is their application in food packaging to protect against faster spoilage [118]. *Lactobacillus* species (LAB) were incorporated into alginate–gelatin fibers, averaging 420 nm in diameter, which is much smaller than described in previous studies. The survival rate of the probiotic bacteria was approximately 96%, although this depends not only on the process conditions but also on the bacterial species studied [119]. In the study, it was observed that the bacterial survival was directly correlated with the cellular hydrophobicity. Hydrophilic bacterial strains demonstrated a greater reduction in viability compared to their hydrophobic counterparts. This characteristic is primarily influenced by factors such as the presence of exopolysaccharides (EPS) and various surface proteins, which can provide essential protection against environmental stresses [120]. Additionally, the strain morphology can also influence post-process survival. *Lactobacillus delbrueckii*, characterized by its larger cell size, may consequently exhibit lower viability compared to other tested strains with smaller cells. Alginate mats with probiotic bacteria delayed the growth of typical pathogenic bacteria in fish fillets. However, a gap in this research lies in the need to investigate the quantity of bacterial cells that are released to determine the optimal bacterial dose required to effectively inhibit pathogen growth [121].

Various electrospinning techniques are employed to encapsulate microorganisms, including single-needle setups, coaxial configurations, and nozzle-free methods. The coaxial method was used, where the core consisted of a mixture of *Lactococcus lactis* 11,454 bacteria and alginate, while the outer shell was composed of a mixture SA/PEO/polysorbate 80 (PS80)/CaCO_3_ [122]. CaCO_3_ was specifically incorporated to improve the bacterial viability in acidic environments by neutralizing the surrounding conditions [123]. This strategy resulted in a reduction in bacterial viability by only one logarithmic order compared to fibers without CaCO_3_.

The nozzle-free method, less commonly used than the single-nozzle approach, allows the elimination of issues related to needle clogging [124]. During the immobilization of *Lactobacillus paracasseri* K7 in SA–PEO–inulin matrix, a significantly higher voltage than previously reported was required for the formation of fibers [125]. The incorporation of inulin into a polymer blend has been reported to positively influence the electrospinning process and support the viability of LAB [126]. However, other reports also indicate that the addition of this prebiotic may negatively affect the structure of the obtained fibers, due to increased viscosity and reduced conductivity of the spinning solution, and fail to promote probiotic growth [125].

There are limited studies on incorporating bacteria and microorganisms other than probiotics into electrospun fibers, particularly alginate nanofibers. To explore this area, *Escherichia coli* was employed as a model organism with relatively straightforward genetic manipulation capabilities and a well-characterized metabolism [127]. In such studies, the incorporation of green fluorescent protein (GFP) into *E.coli* enabled precise localization of bacteria within the fibers [109]. This enabled the successful incorporation of bacteria into fibers during post-processing. Another intriguing area of research is the potential application of *Bacillus* species, which can form spores in addition to vegetative cells [128]. These spore-forming bacteria may exhibit significantly higher resilience to harsh conditions associated with the immobilization process, including mechanical stress, dehydration, and exposure to organic solvents. Moreover, bacterial spores exhibit significantly greater and prolonged stability compared to vegetative cells, which underscores their potential for application in products that require an extended shelf-life. This resistance makes them promising candidates for future electrospinning applications, as it enables greater flexibility in process conditions and material modifications [129].

The possibility of incorporating a wide range of microorganisms into electrospun fibers enables the creation of functional materials with applications in medicine, environmental protection, or agriculture. A key parameter is the survival of microorganisms, which has a direct impact on their functionality and effectiveness in practical applications.

**Table 3:** Alginate—based fibers with the addition of microorganisms.

Polymers	Solvents	Surfactant	Microorganism	Electrospinning Conditions	Crosslinking	Application	Results/Conclusion/Problems/Perspectives	Reference
SA, PVA	water	-	*Lactobacillus paracasei* KS-199	U = 22 kV	-	method of probiotic encapsulation; probiotic food	-Bacteria incorporation yielded beaded fibers (diameter increased from 305 nm to 842 nm) -Electrospinning did not remarkably influence *L. paracasei’s* stability or metabolism -Nanofiber mats exhibited high melting temperature, suggesting potential for heat-processed/baked foods.	[114]
				Q = 1.2 mL/h				
				L = 10 cm				
SA, corn starch	water	-	*Lactobacillus acidophilus* LA5, *L. rhamnosus* 23,527 LGG, *Bifidobacterium bifidum*, *B. animalis*	U = 24 kV	freeze-drying after the process	method of probiotic encapsulation; oral consumption of probiotics	-Free nanofibers had a diameter of 295 nm; probiotics-loaded nanofibers had a diameter of 797 nm -Enhanced viability was observed compared to non-immobilized bacteria -Nanocapsules protected probiotic cells from gastric acid and bile salt effects -Electrospinning significantly enhanced the acid tolerance and viability of the tested bacteria. -Optimized methods and biopolymer formulas are needed to improve the survival/viability of probiotic species/strains in digestive juices and expand their food/supplement application.	[130]
				Q = 1.5 mL/h				
				L = 12 cm				
				T = 25 °C				
core: SA/bacteria shell: SA/PEO/CaCO_3_/PS80	water	PS80	*Lactococcus lactis* 11454	U = 17.5 kV	CaCl_2_ in water; CaCl_2_ in 1:1 water/glycerol	delivery system of probiotics to the gut	-Flowing alginate in the core needle helped to reduce interfacial tension variance that can occur where both solutions meet at the coaxial needle tip -CaCO_3_ was mixed into the shell solution to improve the acid survivability of encapsulated bacteria. -Survivability after the electrospinning process was 10^8^ (10 times smaller than the initial ratio) -Release from fiber in simulated gastrointestinal tract model was significantly higher with the addition of CaCO_3_ (no release w/o CaCO_3_) -The antacid enabled the survivability of encapsulated bacteria in the stomach	[15]
				Q= Core: 0.35, Shell:0.70 mL/h				
				L = 18 cm				
				T = 23 °C				
				RH = 20–30%				
SA, gelatin	water	-	*Lactobacillus acidophilus*, *Limosilactobacillus reuteri*, *Lacticaseibacillus casei*, *Lacticaseibacillus rhamnosus*	Q = 0.5 mL/h	-	food packaging	-Encapsulation of probiotics reduced mechanical strength but increased resistance against water -After 14 days at 4 °C, 25 °C, and 37 °C, probiotic survival in nanofibers ranks: *L. acidophilus* > *L. reuteri* > *L. casei* > *L. rhamnosus* (7.37–9.35 log CFU/g) -With a decrease in viscosity, the stabilization of the jet increases -The need to check the release of bacteria over time to determine the required dose to inhibit the growth of pathogens	[118]
				U = 25 kV				
				L = 15 cm				
				T = 25 °C				
SA, PEO + inulin	water	-	*Lactobacillus paragasseri* K7	U = 60 kV	-	delivery system of probiotics (drugs, hygiene products, urogenital infections treatment)	-Addition of inulin caused an increase in viscosity when spinning solutions and the nanofibers were potentially formed only when the solution did not contain inulin -One of the limitations presents the release study where only samples immediately after the performed electrospun process were taken for analysis. These results are not enough to determine the significance of the effect of ageing and storage conditions.	[125]
				L = 21 cm				
SA, PVA + inulin	water	-	*Lactobacillus fermentum*	U= 20 kV	-	delivery system of probiotics	-The formation of beads was observed in the fibers -The addition of inulin influenced the survival of probiotic bacteria -Addition of inulin increased the surface tension of the solution	[126]
				Q = 1.5 mL/h				
				L = 10 cm				
				T = 25 °C				
				RH = 50%				
SA, PEO	water	PS80	*E. coli* with GFP plasmid	U = 17.5 kV	-	medicine- probiotics delivery to the gut	-The addition of surfactant caused the formation of smooth fibers; -The change in conductivity and surface tension of the solution can be significant to produce smooth fibers, especially with the addition of microorganisms	[109]
				Q = 2 mL/h				
				L = 17 cm				
				T = 23 °C				
				RH = 20–30%				
SA, PEO	water	-	*Bacillus* strains	U = 15–17 kV	-	wound healing	-Nanofiber diameters in all prepared formulations ranged from 200 to 300 nm -60% (in case of PEO/SA 60/40 nanofibers) and 40% (in case of PEO/SA 20/80 nanofibers) of the viable spores were released from spore-loaded nanofibers in the first hour of the release experiment. -The presence of SA and the increase of its content in the electrospinning solution resulted in significantly smaller nanofiber diameters (*p* < 0.05), which can be attributed to the increased charge density in the polymer solution	[129] ^1^
				Q = 0.325 mL/h				
				L = 15 cm				

^1^ L-needle-to-collector distance; Q-feed rate; RH-humidity; T-temperature; U-voltage.

### 4.2. Fibers for Drug Delivery Applications

Electrospun nanofibers offer great potential in medicine due to their porosity, controlled drug release, biocompatibility, and biodegradability [131]. They are widely studied in tissue engineering, wound healing, and drug delivery, with the existing research being focused on optimizing their structure and functionality [132]. A key advantage is the ability to incorporate poorly soluble drugs, which improves their bioavailability and enables controlled release [133]. Drug release from nanofibers is typically diffusion-driven [134] and can be tailored for immediate or prolonged action, including burst, sustained, or stimulus-responsive profiles [11].

Various drugs, antibiotics [135,136], anti-inflammatory agents [137,138], and others [139] have been successfully incorporated into electrospun fibers (Table 4). The incorporation of drugs and their subsequent release depend, among other factors, on the type of polymer used. SA–PEO fibers typically exhibit a rapid release of incorporated compounds, which is often attributed to the hydrophilic nature of both polymers and the porous structure of electrospun fibers. Additionally, their excellent liquid absorption capacity makes them highly suitable for managing moist wounds. Incorporating antibiotics such as levofloxacin inhibits a broad spectrum of Gram-negative bacteria, including *S. aureus* and *P. aeruginosa* [140]. SA–PEO–soy protein nanofibers were developed with an average diameter of 300 nm, and vancomycin was embedded within their structure [141]. The incorporation of the drug did not cause any morphological changes in the fibers. These alginate-based fibers exhibited antibacterial activity against *E. coli* and *S. aureus* and were confirmed to be non-toxic to HDF (human dermal fibroblast) cells. SA–PVA nanofibers are widely used in wound healing due to their excellent mechanical properties, which are crucial for exudate absorption and maintaining hemostasis [142]. Additionally, through crosslinking of the fibers after electrospinning, a product with enhanced aqueous strength can be obtained [143]. GA, one of the most commonly employed crosslinking methods, was utilized to fabricate SA–PVA fibers with immobilized amoxicillin [142]. However, despite its effectiveness, this method is associated with cytotoxicity towards cells. In this case, the incorporated drug interacted with the polymer matrix through hydrogen bonding, which contributes to the stability of both drug immobilization and the electrospinning process. Alternatively, polydopamine (PDA) was successfully applied, interacting with the functional groups of alginate to form stable chemical bonds [143]. This led to the production of durable, non-toxic, and antibacterial alginate mats with immobilized ciprofloxacin. Ciprofloxacin, known for its antibacterial properties, was incorporated into the electrospinning solution of a SA–PVA fiber with agarose. Agarose, a natural polysaccharide with neutral properties, was investigated for its potential to improve the electrospinnability of alginate. However, despite its promise, agarose was unable to produce smooth fibers and did not enhance the electrospinning process [45]. In contrast, the addition of PAA (polyacrylic acid) to the SA–PVA blend improves fiber absorption, and the incorporation of ciprofloxacin as an active component inhibits the growth of both Gram-positive and Gram-negative bacteria for at least 7 days [60]. The effect of the concentration of the drug incorporated into PVA–SA fibers on the inhibition of bacterial growth, focusing on *P. aeruginosa* and *S. aureus*, was investigated [144], which revealed that the drug amount could be reduced by up to half while still achieving the effective inhibition of bacterial growth. This result suggests that encapsulating drugs within electrospun fibers enhances their antimicrobial efficiency, potentially due to improved localized delivery or sustained release. Furthermore, the fibers obtained in the study had the smallest diameter when the mixture contained 3% alginate. Based on the versatile applications of SA–PVA fibers, incorporating various agents, including antibacterial and antifungal agents such as clotrimazole, is possible. Clotrimazole effectively inhibits the growth of yeasts and fungi, including *Candida* and *Aspergillus*. Clotrimazole-loaded SA–PVA–dextran nanofibers exhibit superior antifungal activity and bioadhesion compared to traditional films, presenting a promising alternative for treating vaginal candidiasis [145].

Drugs immobilized in alginate nanofibers enable targeted delivery and controlled release of the incorporated substance. The potential of this method allows for the design of formulations tailored to the individual needs of patients, which ensures high quality and safety.

### 4.3. Alginate Fibers Modified with Nanoparticles

NPs have garnered significant attention, mainly due to their antibacterial activity, which stems from their interaction with the bacterial cell wall and their ability to intensify oxidative stress by generating reactive oxygen species [150]. Incorporating metallic NPs such as silver and zinc oxide into electrospun alginate-based fibers enhances their properties, making them more suitable for various applications. Table 5 presents a review of several articles that focus on the addition of NPs into electrospinning solutions, summarizing the key conclusions of their research. The incorporation of ZnO into the SA–PEO aqueous electrospinning solution has the potential to improve the resulting fiber’s performance in removing tetracyclines from contaminated water. Increasing the concentration of ZnO NPs in nanofibers enhanced their adsorption capacity. However, loading above 10% led to the accumulation of NPs on the fibers, which blocked the exposure of active sites and reduced the adsorption efficiency. The produced nanofibers (100 mg) removed over 99% of tetracyclines from 5 mL water samples containing 10 mg/l of tetracyclines within 120 min, which confirmed good adsorption [151]. ZnO NPs combined with *Salvia abrotanoides* oil enhanced the antibacterial and wound-healing effects of the fibers. The addition of NPs improved the mechanical properties of fibers, such as their tensile strength and elasticity. However, the authors limited their evaluation to numeric values without providing an analysis or discussion of the mechanisms by which the NPs influenced the mechanical properties. Also, the addition of NPs increased the antimicrobial activity against *S. aureus* and *E. coli*, promoting the proliferation, attachment, and viability (>90%) of L929 cells. Complete wound healing was observed within 14 days, which is superior to the performance of unmodified scaffolds, which, after 14 days, exhibited an incomplete epidermal layer, reflecting poor wound repair [152]. Alginates with low molecular weight and high mannuronic acid content favor interactions with ZnO due to containing more exposed carboxyl and hydroxyl groups [65].

The addition of Ag was examined for biomedical and food packaging applications. Membranes turned out to be stable in aqueous suspension for months, which indicates that SA is an effective protecting agent for Ag colloids. Positive stability tests along with the antibacterial properties of Ag indicate that the resulting fibers are exploitable for these purposes [51].

The incorporation of NPs into electrospun alginate-based fibers enhances their properties, making them a good candidate for water purification and wound-healing applications. The modified fibers exhibit improved performance in terms of their adsorption and antibacterial effects, which expands their potential uses.

### 4.4. Incorporation of Plant Extracts into Alginate Fibers

Plant extracts and essential oils are increasingly incorporated into alginate fibers to boost their antioxidant, antimicrobial, and anti-inflammatory properties for biomedical and packaging applications [152]. Table 6 presents an overview of recent studies that demonstrate the applications and enhanced functionalities of alginate-based electrospun fibers when active compounds from plant extracts and essential oils are added. Curcumin, added to SA–PEO fibers and crosslinked with TFA, enhanced the tensile strength and stiffness of the fibers, though it reduced their flexibility, probably because of the suppression of the PEO after the crosslinking process. While curcumin concentrations of up to 10% slightly enhanced the mechanical performance, higher concentrations negatively impacted the fiber’s properties due to a higher concentration of defects and the loss of the nanostructure of the mat. Therefore, curcumin does not have a significant effect on mechanical strength compared to the crosslinking process [41]. The researchers employed an interesting approach, developing SA–PVA films with the addition of black wolfberry anthocyanin (BWA) for food packaging applications. As the BWA content increased from 0% to 5%, the mechanical performance of the films improved due to hydrogen bonding between the BWA and the polymer matrix components, which enhanced the crosslinking network density between biopolymers. However, with further increases in the BWA concentration, excessive anthocyanins disrupted the polymer network, which resulted in a decrease in mechanical properties. This also affected the film’s hydrophobicity. As the BWA concentration increased, the hydrophobicity improved due to a reduction in free hydroxyl groups, which were involved in hydrogen bonding between the BWA and the polymer [41]. Anthocyanins, extracted from purple cabbage (*Brassica oleracea* var. *capitata*), were added to an SA–PVA electrospinning solution to produce pH-sensing electrospun mats. These mats effectively monitor pH changes in open wounds, which makes them suitable for wound-healing applications. The results showed that the pH-detecting mat exhibited distinguishable color changes at different pH levels, except for the pH range of 6.0–8.0. The mat responded quickly when tested on an open wound, with visible color changes despite limited exudate. Its healing performance matched that of commercial dressings, highlighting its promise for advanced wound care [154]. Researchers also focused on enhancing wound-healing properties by using bioactive compounds. Adding *Lawsonia inermis* and *Scrophularia striata* extracts resulted in mats with an antibacterial rate that was approximately 70% higher than those without the extracts. However, as the concentration of the extracts increased, the tensile strength decreased due to interactions that increased entanglement and mobility of the polymer chains. Mats with *Lawsonia inermis* extract resulted in a 45% faster wound-healing process than the control, while mats containing *Scrophularia striata* extract showed a 40% improvement [152].

In response to the growing antibiotic resistance of bacteria, the use of natural extracts is emerging as a promising strategy in wound healing. Alginate–PEO fibers impregnated with oregano oil exhibited potent antibacterial activity against MRSA (methicillin-resistant *Staphylococcus aureus*), which makes them suitable for advanced wound dressings [155]. The SA–PEO blend was used as a coating, together with embelin—a natural benzoquinone derivative with anti-inflammatory and antibacterial properties, and 3-hydroxybutanoic acid (PHB)—a polymer known for its excellent mechanical properties when combined with other polymers. However, the authors only mention these potential benefits and do not investigate the influence of interactions between the polymers on the mechanical strength of the resulting fibers, nor do they evaluate their mechanical performance [156].

The wide range of potential applications highlights the adaptability of plant extracts and underscores the need for continued exploration of their properties and practical applications.

**Table 6:** Alginate-based fibers with added extracts.

Polymers	Solvents	Surfactant	Active Component	Electrospinning Conditions	Crosslinking	Application	Results/Conclusion/Problems/Perspectives	Reference
SA, PEO	water	-	curcumin	L = 15 cm U = 15–23 kV Q = 0.3–1 mL/h t = 3 h Room temperature RH = 40–50%	TFA	environmental pollution control, food packaging and tissue engineering	-TFA crosslinking and the addition of curcumin increased the mechanical properties of the fibers. -Curcumin content up to 10% increased mechanical strength; higher concentrations have a negative effect	[41]
SA, PVA	water	-	purple cabbage anthocyanins	L = 12 cm	GA	tissue engineering	-Uniform nanofibers were obtained with proper incorporation of the extract into the nanofibers and produced mats worked well for monitoring pH -Psychochemical properties of polymer solution strongly affect the morphology of fibers; addition of PVA increases the conductivity and disruption of the jet structure	[154]
				Q = 0.7 mL/h				
				U = 18 kV (without extract)				
				U = 14 kV (with extract)				
SA, PVA	water	-	BWA	Q = 0.5 mL/h	-	intelligent packaging	-As anthocyanin concentration increased, fiber diameter and tensile strength decreased, while thickness, moisture content and antioxidant properties increased -High sensitivity to the pH environment- color changes -Formation of the beads and agglomerates due to increased viscosity of the solution, due to the addition of BWA, which affected the fiber structure and diameter	[157]
				U = 20 ± 0.5 kV				
				L = 10 cm				
				T = 25 °C				
				RH = 50%				
SA, PVA	water	-	*Lawsonia inermis* and *Scrophularia striata* extracts	U = 10 kV	GA	biomedical application—wound healing	-As the concentration of the extracts increased, the tensile strength decreased -Extracts caused faster wound healing -One limitation is the need for further investigation into the long-term biocompatibility and degradation	[158]
				Q = 0.4 mL/h				
				L = 12 cm				
SA, PCL	water, chloroform, methanol	-	*Salvia abrotanoides* essential oil	U = 25 kV L = 15 cm shell solution: Q = 0.2 mL/h core solution: Q = 1 mL/h T = 25 °C RH = 30%	-	tissue engineering—wound healing	-The average diameter of nanofibers: 187 ± 51 nm, improved tensile strength -Application of ZnO NPs and essential oils increased antimicrobial activity -The scaffold accelerated the healing time with total wound healing over 14 days in mouse models carrying full-thickness wounds compared to the nanofibrous scaffold without additives. -While the drug release profile of the membrane was satisfactory, close to 50% of the bioactive agents were not released in 5 days	[152]
SA, PVA	water	-	*Malva Sylvestris* extract	T = 25 °C	GA	tissue engineering—wound healing	-Diameter ranged from approximately 100–200 nm, without beads -At 21st day of treatment SA/PVA/extract dressing demonstrated a wound closure rate of 93% while the control (without an extract: 60%)	[159]
				Q = 0.4 mL/h				
				U = 12 kV				
				L = 12 cm				
SA, PEO, poly(3-hydroxybutyric acid)	water, ethanol, hexafluoro-2- propanol,	-	embelin	Q = 2 mL/h electric potential: 1 kV/cm L = 15 cm	GA	tissue engineering—wound healing	-Coaxial mats showed antibacterial properties (because of the addition of embelin), improved physicochemical properties -Core-shell structure also ensures a sustained release profile -A limitation of the study is that the manufacturing techniques for nanofibrous mats may not be able to produce mats that are large enough or contain the structural integrity to treat deep wounds effectively	[156]
SA, bentonite (clay)	water	-	shrimp enzyme extract	Q = 10 mL/h	CaCl_2_ (collector solution) with or without chitosan in aqueous or acetic acid solutions	feeds for aquaculture	-The average sizes ranged from 360 to 790 μm (wet) and 260–516 μm (freeze-dried), with larger sizes for those including chitosan and bentonite -SA–chitosan–bentonite was the most suitable for immobilizing exogenous proteases -Clay sonication pre-treatment can prevent mineral agglomeration and achieve better dispersion within the composite matrix. This method not only enhances barrier properties but also helps preserve the enzymes	[160]
				U = 15 kV				
				L = 20 cm				
SA, PVA	water	-	cardamom extract	U = 15 kV	GA	tissue engineering—wound healing	-Optimum content of extract was 10 wt%, resulting in fibers with average diameter of 233 ± 33 nm. -Nanofibers showed no toxicity against fibroblast cells -Increasing the amount of PVA in the blend solution reduces the surface charge density of SA, leading to the formation of a thicker jet during the electrospinning process - When L is too short, beads form because the jet lacks sufficient time to stretch properly, and the diameter of fibers increases	[161]
				Q = 1 mL/h				
				L = 10 cm				
SA, PVA, Poly-(D, L,-O-lactide)-co-glycoside	SA, PVA: water Poly-(D, L,-O-lactide)-co-glycoside: chloroform, DMF	-	*Capparis sepiaria* plant extract	Q = 10 µL/min	-	tissue engineering—wound healing	-Exhibited smooth and porous surfaces, and showed high % cell viability of 90% and above, revealing non-toxicity and biocompatibility -Poly-(D, L,-O-lactide)-co-glycoside addition enhanced the surface charge density and conductivity of the polymer solution, increasing the jet’s stretching force and leading to nanofibers with a smaller diameter	[162]
				U = 15 kV				
				L = 15 cm				
SA, PEO	water	Pluronic F-127	OEO	U= 26 kV; 30 kV Q= 0.8 mL/h L= 15 cm Room temperature RH = 40–60%	CaCl_2_ for 10 min; dH2O for 23 h	medical textiles	-Addition of OEO caused lower viscosity -The smallest beads were obtained with the applied voltage of 26 kV -Without soaking in water for 24 h—fibers were embedded in PEO film layers; the same sample, but with the additional step of soaking in water for 24 h, showed clear fibers, without PEO -Mats had antimicrobial activity against MRSA -The crosslinking process is necessary to prevent the SA nanofibers from dissolving in water for their end-use	[155] ^1^

^1^ L-needle-to-collector distance; Q-feed rate; RH-humidity; T-temperature; U-voltage.

## 5. Commercialization of the Electrospinning Process

### 5.1. Alginate Electrospinning Patents

The growing number of patents reflects the rapid development of alginate electrospinning technology. As of March 2025, Espacenet lists nearly 2500 patents related to alginate electrospinning, with 151 patents identified solely by their titles. These inventions are applied in the medical, pharmaceutical, and food sectors. Table 7 presents selected patents that provide a detailed description of the methodology for producing electrospun fibers based on a SA solution.

**Figure 5:**
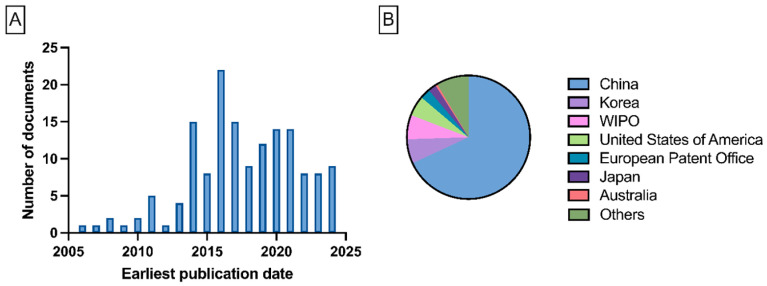
The number of patent applications related to alginate electrospinning based on the Espacenet data base (ti = “alginate” AND (nftxt = “electrospinning” OR nftxt = “electrospun”); (**A**): number of patents by year; (**B**): patent applications by country.

China leads the global patent activity, with contributions from both academia and industry (Figure 5). Patents frequently describe the blending of alginate with PEO, gelatin, and additives such as surfactants or plasticizers to enhance its spinnability and material performance. Moreover, the available patents provide detailed descriptions of the crosslinking methods employed, including thermal and chemical approaches. An interesting approach involved the development of a fluid electrospinning method, where alginate fibers fell into the chitosan solution together with Ca^2+^ ions, which enabled immediate crosslinking [163]. The patents cover uniaxial, coaxial, needleless, and fluid electrospinning techniques. The patent activity confirms the rapid evolution of alginate electrospinning, with growing interest in novel materials and methods, particularly for biomedical applications.

**Table 7:** Patents related to the production of electrospun alginate fibers (search results from Espacenet: ti = “alginate” AND (nftxt = “electrospinning” OR nftxt = “electrospun”)).

Name	Number	Short Description	Origin	Reference
Nano fibrous frame material with sodium alginate as matrix and its preparation method	CN100443126C	The patent provides a method for producing nanofibers based on SA/PVA or SA/PEO, with the addition of glycerin and a surfactant. The invention also describes a chemical crosslinking process.	China	[164]
Method for preparing pure sodium alginate nano fiber membrane material	CN101230150A	The patent describes an electrospinning process of pure SA dissolved in a water-ethanol solution, performed by fluid electrospinning.	China	[165]
Alginate-based nanofibers and related scaffolds	US2009087469A1	The patent provides a method for producing nanofibers based on a SA–PEO solution with the addition of a surfactant and a co-solvent (DMSO) to improve the spinnability of alginate.	United States of America	[166]
Method for preparing sodium alginate-chitosan nano-grade medical dressing	CN104069536A	The patent provides a method of producing nanofibers based on a solution of SA–co-polymer (e.g., PVA, PEO). Process is performed by fluid electrospinning, where a receiving solution is chitosan with a crosslinking agent (e.g., CaCl_2_)	China	[163]
Method for preparing sodium alginate electro-spinning nanofibers by chemical crosslinking	CN106222798A	The patent describes a method for producing nanofibers using a SA solution, with epichlorohydrin added to achieve chemical crosslinking of the alginate.	China	[167]
Preparation method of sodium alginate/polyvinyl alcohol nanofiber	CN106283269A	The patent describes an electrospinning method based on a SA–PVA solution.	China	[168]
Antibacterial liquid-absorption type sodium alginate-based composite nanofiber medical dressing	CN108837179A	The invention provides an antibacterial composite nanofiber based on SA–PVA solution enriched with a cationic polymer. The patent also describes a thermal crosslinking method.	China	[169]
Chitosan/calcium alginate needleless electrospinning nanofiber membrane for medical dressings and preparation method thereof	CN109267240A	The patent provides a method for producing nanofibers based on SA–PVA or SA–PEO solutions with the addition of a chitosan–PEO composite solution, performed using needleless electrospinning.	China	[170]
Preparation method of sodium alginate and chitosan composite nanofiber	CN111020745A	The invention describes a method for producing nanofibers based on a SA–chitosan solution.	China	[171]
Porous sodium alginate nanofiber scaffold material and preparation method thereof	CN113577397A	The patent provides a method for producing nanofibers based on a SA–PVA solution. The invention describes a foaming and crosslinking process to obtain a porous material.	China	[172]
Chitosan-alginate composite nanofiber membrane, as well as the preparation method and application thereof	CN112760811A	The patent provides a method for producing nanofibers based on a SA–chitosan–gelatin solution, incorporating drying.	China	[172]
Alginate-based fibers and uses thereof	US2022033995A1	The patent describes a method for producing nanofibers based on SA–cellulose with the addition of CaCl_2_.	United States of America	[173]
OMVs-loaded sodium alginate nanofiber membrane, as well as the preparation method and application thereof	CN118480968A	The patent describes a method for producing nanofibers based on a PVA–SA solution loaded with OMVs.	China	[174]

### 5.2. Electrospinning Process on an Industrial Scale

Depending on the production requirements, manufacturers offer electrospinning set-ups tailored to the process needs. With the growing interest in laboratory-scale electrospinning, competition among equipment suppliers has intensified significantly. As a result, the market now offers a wide range of pilot- and industrial-scale systems, along with specialized accessories [175]. Scaling up remains challenging due to lower productivity and time-intensive optimization [176]. The primary features considered when choosing these devices are their price and multifunctionality. At the laboratory scale, manufacturers typically offer needle-type electrospinning setups. The electrospinning process, on an industrial scale, is most often carried out using a rotating drum to collect the fibers. When designing the process for the selected set-up, attention is paid to features such as the equipment productivity, the most significant possible process control capacity, and quality control of the produced fibers [175].

Manufacturers offer diverse, multifunctional setups to meet market demands, which results in a broad range of equipment availability. Table 8 lists selected manufacturers of electrospinning equipment that provide products for conducting the process on an industrial scale. Elmarco company offers an industrial-scale electrospinning device that utilizes the needleless method. Nanofibers are created using the Nanospider^TM^ technology (Liberec, Czech Republic), a process that utilizes thin wire-shaped electrodes that are continuously coated with a polymer. High voltage between two electrodes allows for the formation and simultaneous collection of fibers. SKE Research *Equipment* also provides software for its electrospinning machines, allowing the process to be controlled remotely. This solution allows for continuous processes without the need to interrupt work. During the electrospinning process using needle methods, the number of fibers is crucial to ensure the process’s optimal efficiency. Tong Li Tech (Shenzhen, China) offers a device equipped with a multi-needle spinneret that can hold up to 256 needles simultaneously. Additionally, the device features an online cleaning option, which automatically cleans the needles, greatly simplifying the process when using multiple solutions.

Electrospinning’s low single-needle yield (often only ~1–100 mg/h) poses a major scale-up challenge [177]. To industrialize alginate nanofiber production, multi-jet strategies have been adopted; among them, multi-needle arrays and needleless free-surface systems can raise the output by producing many jets in parallel. For example, an industrial setup can reach hundreds of grams per hour of nanofiber production, which is orders of magnitude above lab-scale rates [178,179]. These methods substantially reduce the unit production cost of fibers; however, the associated requirements for the high-voltage power supply and large-scale solvent management increase proportionally with the production capacity. Notably, electrospinning does not demand high heat or pressure, so its energy consumption per gram is moderate [180]. Recent engineering innovations show that this is a feasible redesign of a concentric spinneret, with the energy usage reduced to only 54% of a standard setup’s consumption [181]. When integrated with continuous operation and solvent recovery strategies, such technological advances contribute significantly to bridging the gap toward economically viable, industrial-scale production of alginate nanofibers. Electrospinning of alginate has advanced beyond laboratory-scale feasibility; however, achieving an industrial cost-effective output will require further optimization of the process’s throughput, energy efficiency, and fiber uniformity.

Electrospinning is a highly efficient technique for creating nanofibers, which is why market competition is constantly growing. The search for new solutions and modifications to existing methods has a significant impact on the development of technology, enabling the creation of more versatile and efficient devices that are optimal for industrial applications.

**Table 8:** Selected manufacturers of industrial-scale electrospinning equipment.

Company	Set-Up Scales Available	Reference
Elmarco (Liberec, Czech Republic)	laboratory and industrial	[182,183]
SKE (Research Equipment) (London, UK)	laboratory, pilot, industrial	[184,185]
Nano FiberLabs (Foshan, China)	laboratory, pilot, industrial	[186]
Electrospinning (Didcot, UK)	laboratory, pilot, industrial	[187,188]
Fnm Co. (Milan, Italy)	laboratory, pilot, industrial	[189,190]
INOVENSO (Cambridge, MA, USA)	laboratory, pilot, industrial	[191,192]
Nanoflux (Singapore)	laboratory, pilot, industrial	[193]
Fluidnatek by Bioinica (Valencia, Spain)	laboratory, industrial	[194,195]
Progene Link Sdn Bhd (Subang Jaya, Malaysia)	laboratory, pilot, industrial	[196]
NanoLab (Waltham, MA, USA)	laboratory, pilot, industrial	[196]

## 6. Research Directions and Knowledge Gaps

Despite growing interest and substantial progress in the electrospinning of alginate, several scientific and technological gaps remain that need further investigation. These gaps span from fundamental polymer science to application-specific design and process engineering.

1. Systematic studies on the source, purity, and molecular characteristics of alginate need to be carried out. Many research papers neglect the critical role of raw material variability in the electrospinning process. Alginates from different suppliers, or even from different production batches, can vary significantly in their molecular weight, block composition (M/G ratio), and viscosity. These differences directly affect solution behavior, jet stability, and fiber formation. Comparative studies using alginates of various origins and properties, processed under identical conditions, are urgently needed. Developing standardized protocols for polymer characterization will help define guidelines for selecting alginates that are suitable for fiber production.
2. The development of fully biodegradable systems without synthetic polymer carriers is a necessity. Currently, most alginate electrospinning methods require the use of synthetic polymers such as PEO or PVA to facilitate fiber formation. Although these materials improve processability, they reduce the biodegradability of the final product and pose obstacles for sustainable development and green manufacturing. Future research should focus on creating entirely natural systems using only biopolymers, such as pullulan, chitosan, or gelatin, combined with alginate. The goal is to develop universal and scalable electrospinning methods that eliminate synthetic additives while maintaining the quality of the fibers.
3. Interaction of process parameters with modified alginate systems: While the spinning conditions (voltage, distance, temperature, humidity) are well described for standard blends, these blends may behave differently when novel, chemically modified, or biopolymer-only alginate systems are used. There is a need for integrated studies that couple formulation chemistry with systematic process optimization (i.e., response surface methodology) methodologies.
4. Fiber structure-function correlations for targeted applications: Many studies focus on describing the morphology and mechanical properties of alginate-based fibers, but often overlook how these features impact their practical performance. To design materials suited for specific applications, it is essential to understand how the fiber’s structure, including its porosity, surface area, and crosslinking, relates to key functional outcomes, including drug release, microbial survival, and gas permeability. Future research should include standardized tests that directly measure these functions to help connect the structure of fibers with their real-world performance.
5. Evaluation of long-term stability and degradation in real-use conditions: Current studies typically focus on short-term fiber stability, which is usually measured in hours or days. However, real-world applications require predictable behavior over weeks or months. This is critical for wound dressings, agricultural mats, packaging materials, and filters. Long-term studies should assess aging, moisture uptake, degradation kinetics, and storage stability. Furthermore, it is essential to understand how crosslinked alginate nanofibers behave in biological and environmental systems to ensure safe and environmentally friendly degradation.
6. Investigation of interactions between the electrospinning process and living microorganisms: Encapsulating live microorganisms into nanofibers is an emerging and promising direction; however, there is limited knowledge about how this process affects cell viability and function. Research is needed to study how shear forces, electric fields, and osmotic pressure changes during electrospinning impact cell membranes, gene expression, and metabolic activity. Long-term functional studies should assess whether microbes retain their properties, including probiotic activity, biofilm formation, and enzymatic degradation abilities, after encapsulation.
7. Development of greener, safer solvent systems for electrospinning: Although alginate is often electrospun from aqueous solutions, blending it with PEO or PVA typically requires additional solvents or post-processing steps that may involve organic compounds. There is a growing need to develop non-toxic, environmentally friendly solvent systems that are suitable for large-scale production. Ethanol–water mixtures, natural deep eutectic solvents, or enzymatic modification techniques could provide promising alternatives.
8. To enable industrial implementation, it is necessary to develop standardized protocols for the reproducible and scalable production of alginate nanofibers. This includes solving issues related to quality control, real-time process monitoring, and safe handling of materials. Collaboration with industry partners will be crucial to adapting laboratory methods for large-scale production while meeting regulatory, economic, and environmental requirements.

## 7. Conclusions

Alginate is a biopolymer of considerable interest due to its biocompatibility, biodegradability, and functional versatility. Nevertheless, its direct application in electrospinning remains challenging, primarily because of its rigid molecular structure, high viscosity, and polyelectrolytic behavior, which hinder the formation of uniform nanofibers. To overcome these limitations, alginate is typically blended with synthetic or natural copolymers, chemically modified, or processed with surfactants to enhance its spinnability and fiber-forming properties. While the majority of studies have concentrated on biomedical applications, including wound dressings, tissue engineering, and drug delivery, alginate-based nanofibers also show promise in non-medical domains such as agriculture, water purification, and active food packaging. An emerging area of interest involves the incorporation of living microorganisms into alginate fiber matrices, which has potential applications in probiotic delivery, microbial immobilization, and environmental bioremediation.

Despite these advances, several critical research gaps persist. There is a need to develop fully bio-based, synthetic-free electrospinning systems to better understand the structure–function relationships in alginate fibers and to investigate their long-term stability and performance under application-relevant conditions. Addressing these challenges will be essential for the broader adoption of alginate nanofibers in sustainable and high-performance material technologies.

## Figures and Tables

**Table 1:** Summary of the properties of alginates used for electrospinning.

Molecular Weight kDa	Reference	M/G Ratio	Reference
<40	[41]	0.42	[42]
66	[43]	0.42	[44]
80–120	[45]	0.43	[46]
115	[47]	0.43	[34]
130	[48]	0.54	[49]
138	[50]	0.64	[51]
140	[47]	1.05	[52]
144–148	[50]	1.25	[53]
158	[43]	1.25	[54]
120–200	[55]	1.25	[56]
120–200	[57]	1.25	[58]
120–190	[59]	1.30	[60]
150–180	[61]	1.42	[62]
200	[63]	1.49	[64]
200	[51]	1.56	[25]
220	[52]	1.56	[65]
220	[66]	1.56	[8]
323	[58]	1.56	[67]
323	[68]	1.56	[69]
324	[53]	1.56	[70]
396	[64]	1.56	[71]
486	[50]	1.61	[43]
500	[25]	2.33	[72]
500	[65]	2.65	[73]

**Table 2:** Alginate-based fibers without the addition of active components.

Polymers	Solvents	Surfactant	Electrospinning Conditions	Crosslinking	Application	Results/Conclusion/Problems/Perspectives	Reference
SA, PVA	water	-	U = (1;15.50;30 kV)	GA	skin tissue engineering	-Optimal solution: SA:PVA 1:6,5 nanofibers with regular size and narrow diameter (<170 nm) were obtained at 15–30 kV, 0.55–1.00 mL/h and 12.5–20.0 cm -Pure PVA was slightly toxic and irritant for surrounding tissues; its biocompatibility can be improved with the addition of natural polymers	[75]
			L = (5;12.50;20 cm)				
			Q = (0.1;0.55;1 mL/h)				
SA, PLA	water, chloroform	Span 80	Q = 0.5 mL/h	CaCl_2_	tissue engineering	-Emulsion electrospinning is a good method to produce fibers with good mechanical strength (only with crosslinking)	[76]
			L = 15 cm				
			U = 15 kV				
SA, PEO	water, ethanol	Triton X-100	L = 20 cm	CaCl_2_	wastewater treatment	-Solution of electrospun SA/PEO fibers in water mixed with chitosan. Produced composite sponges have potential in an adsorption	[77]
			U = 15 kV				
			T = 25 °C				
			RH = 30%				
SA, PEO	water, ethanol	Triton X-100	Q = 0.30 mL/h	CaCl_2_/TFA/GA	water treatment	-Different crosslinkers could adjust the efficient adsorption at varied environmental conditions -CaCl_2_-crosslinked membranes showed the best tensile strength, and TFA-crosslinked membranes had the highest specific surface area.	[58]
			L = 15–20 cm				
			U = 20–28 kV				
			T = 30–35 °C				
			RH = 30–35%				
SA, PEO	water	PS80	Q = 2 mL/h	CaCl_2_ in different environments	biomedical application	-In each case, crosslinked fibers did not dissolve in water as uncrosslinked fibers did and retained their morphology -fibers are more stable in the acidic gastric modeling environment -Ethanol is not ideal for the encapsulation/delivery of biologics, as it can cause cell death and promote oxidative stress that damages proteins and DNA -Presence of glycerol in calcium alginate microbeads (presence in solvent mixture) improved their stability and extended the release time -Fibers crosslinked in neutral and basic solutions were not able to maintain their fibrous structure after submersion in PBS for 2 h -The stability of the fibers in acidic environments and their swelling in higher pH environments can be beneficial for the targeted release of active ingredients	[73]
			U = 17.5 kV				
			L = 18 cm				
			T = 25 °C				
			RH = 20–30%				
SA, PEO, methacrylated gelatin	water	F127	Q = 0.1 mL/h	CaCl_2_, UV	tissue engineering	-In addition to Ca^2+^, fibers were also crosslinked by UV, which further stabilized the gelatin -The fibers promoted the adhesion and proliferation of mesenchymal stem cells for 5 weeks -The solvent/polymer interactions provide the surface tension, conductivity and viscosity of the solution as the primary factors that influence electrospinning -The low surface tension of the nonsolvent ethanol used in the bath prevented fiber from dense packing, thus allowing the generation of a 3D macroporous structure which favors cell motility	[64]
			U = 7 kV				
			L = 7 cm				
SA, PEO, PCL	water, chloroform, DMF	Tween 80	U = 29 kV	CaCl_2_	tissue engineering and biomedical applications	-Core-shell fibers showed better mechanical strength than SA-PCL fibers -SA was modified with heat treatment to decrease the molecular weight; (better than degrading the molecule)	[78]
			L = 13 cm				
			Q = 1 mL/min				
SA, pollulan	water	-	free-surface electrospinning RH = 30–33% T = 23 °C U = 30 kV V of carriage contains polymer solution: 80 mm/s Distance from grounded electrode to wire electrode: 18 cm	CaCl_2_ was added to the solution, not after electrospinning	food, nutraceutical, and pharmaceutical applications	-Addition of CaCl_2_ (up to 0.045%, *w*/*w*) resulted in smooth fibers that were smaller in diameter and more thermally stable than those without the addition of CaCl_2_ -The branched fibers are caused by the electric field induced by the excess charge carried on the primary jet -Promising usage as an active food packaging material (w/o ethanol)	[56]
SA, PEO, chitosan	water, HAc, ethanol	-	U = 27 kV	-	pharmaceutical and biomedical aaplications	-Core-shell fiber diameter: 154 ± 35 nm -The fiber structure was preserved even after 24 h in water -When the distance between the needle tip and the collector decreases, a greater number of defects in the membrane appear	[79]
			L = 10 cm				
			Q = 0.4 mL/h				
PVA+SA/KA/LiA	water	-	U = 20 kV L = 15 cm Q = 0.2 mL/h Room temperature RH = 25%	-	without a specific application	-Too much alginate causes bead formation -Substitution of Li^+^ as a hard acid, instead of Na^+^, causes a stronger interaction with the hard base of the carboxylate anion in the alginate backbone and weakens the intramolecular hydrogen bonds and electrospinnability of alginate -Li cation improves electrospinnability and the problem is not in the viscosity (because Li salts have the highest viscosity of the alginate salts) -The results have indicated that the effect of conductivity on the nanofiber diameter is more dominant than solution viscosity	[57]
SA, PEO polypyrrole film (not in the solution)	SA/PEO: water Polypyrrole: ammonium persulfate	-	NEAR-FIELD ELECTROSPINNING L = 1 mm U = 0.8 kV Room temperature	CaCl_2_	tissue engineering	-Polypyrrole is a suitable substrate for cell growth, and alginate is a material unfavorable for cell adhesion, so the alternate arrangement of these materials (contrasting pattern) allows precise cell positioning -By adjusting the level of ionic crosslinking, the stability of alginate fibers can be adjusted, allowing the fibers to initially guide cell positioning and then gradually degrade, creating more space for cell growth	[80]
SA, PCL, carboxymethyl chitosan	water, 1,1,1,3,3,3-hexafluoro-2-propanol,	Span 80	Q = 0.6 mL/h	CaCl_2_	periosteal tissue engineering	-Micron-fibers showed an average diameter of 2.381 ± 1.068 μm with excellent tensile strength and biocompatibility -The addition of the hydrophilic carboxymethyl chitosan and SA could form highly entangled polymer molecular chains and affect the evaporation rate of the solvent. Thus, the surface of the fibers becomes relatively smooth after the addition of carboxymethyl chitosan and SA -The addition of the emulsifier affected the diameter of the fibers	[32]
			U = 16 kV				
			L = 15 cm				
			T = 25 °C				
			RH = 55%				
SA, PEO	water, NaCl	Triton X-100	Q = 0.75 mL/h	SrCl_2_	tissue engineering	-Three alginates (i.e., *M.pyr, L.hyp, A.nod*) were evaluated -Fibroblasts and keratinocytes did not show any preference for the three tested mats, but the former presented a much greater adhesion -Alginate with an evident polyelectrolyte nature, are found to promote cell viability better -Domination of G blocks and high Mw increase charge density, enhancing polyelectrolyte behavior and forcing chains into a rod-like conformation, which reduces viscosity-concentration scaling factors	[50]
			L = 15 cm				
			U = 12.5 kV				
			T = 25 °C				
			RH = 50%				
camphorsulfonic acid-doped alginate-poly(aniline), PVA	water	-	n.a.	GA, CaCl_2_ (double crosslinking)	tissue engineering	-The contact angles of the developed scaffolds with water drop were obtained as 51 ± 2.4° and 58 ± 2.7°, which confirm their excellent wettabilities for tissue engineering applications -The scaffolds have acceptable cytocompatibilities and could improve the MG-63 cells’ adhesion and proliferation -The porous morphologies of both samples may originate from the freeze-drying process	[71]
SA, PEO, collagen, exopolysaccharide	water, HAc	-	L = 15 cm 100 rpm Collagen: Q = 0.5 mL/h U = 17 kV SA/PEO: Q = 0.5 mL/h U = 27 kV collagen/exopolysaccharide: Q = 1 mL/h U = 15 kV	GA	tissue engineering	-Among the nanofibrous scaffolds, Collagen-SA/PEO + exopolysaccharide 2% nanofiber showed better mechanical properties and cellular behavior than others -The high porosity of nanofibers can negatively impact their mechanical properties	[81] ^1^

^1^ L-needle-to-collector distance; Q-feed rate; RH-humidity; T-temperature; U-voltage.

**Table 4:** Alginate-based fibers with added drugs.

Polymers	Solvents	Surfactant	Drug	Electrospinning Conditions	Crosslinking	Application	Results/Conclusion/Problems/Perspectives	Reference
SA, PEO, isolated soy protein	water, NaOH (adjusting pH)	-	vancomycin antibiotic	U = 15 kV	CaCl_2_	biomedical applications	-Smooth and homogeneous SA/PEO/protein fibers with an average diameter of 200 nm were formed -The biocompatibility and non-toxicity of the fibers were confirmed by a cytotoxicity test using human fibroblasts -Increased alginate content showed a negative impact on spinnability and obtained fiber morphology (due to low spinnability of alginate)	[141]
				L = 15 cm				
				Q = 0.5 mL/h				
PVA, SA, PAA	HAc	-	ciprofloxacin (antibiotic)	U = 25 kV	-	tissue engineering—wound healing	-Ciprofloxacin release at neutral pH was approximately 40% and remained constant over 24 h -Fiber diameter of 141 ± 53 nm -PVA–SA–PAA–ciprofloxacin could release drugs for a prolonged period due to their lowest swelling ratio -Controlled release of ciprofloxacin enabled growth inhibition of bacteria for at least 7 days	[60]
				L and Q not specified				
SA, PEO	water, DMSO	Triton X-100	forsythin, CPDs	L = 17 cm	CaCl_2_	tissue engineering—wound healing	-Membranes showed antibacterial and antioxidant properties -Addition of CPDs improved the mechanical strength of membranes -CPDs increased the conductivity of solutions, which allowed the production of nanofibers with a high SA amount (up to 92%)	[146]
				U = 16–20 kV				
				Q = 0.5 mL/h				
SA, silk fibroin	water	-	bornyl acetate (in ethanol)	Q = 0.8 mL/h	ZnCl_2_	medicine- enhanced ulcerative colitis therapy	-Microsphere systems can protect bornyl acetate (the anti-inflammatory drug) from gastric degradation, ensuring its delivery to the colon -Introduction of protein polymers, such as silk fibroin, increases the bioavailability of the matrix—more protein binding sites	[147]
				U = 16 kV				
SA, PVA	water	-	ciprofloxacin, ZnO NPs, dopamine	U = 10 kV	SA/PVA: GA SA/PVA/dopamine: ammonia solution	tissue engineering—wound healing	-The drug release and antibacterial assays demonstrated pH-responsive behavior, with increased drug release under higher pH conditions (simulating bacterial invasion) and strong antibacterial activity, achieving up to 99% inhibition -The burn wound model in rats, nanofibrous mats displayed excellent wound-healing ability in wound closure and tissue regeneration. -With the increase of time exposure to dopamine, the porosity of mats reduces, because of the hydrophilic properties of SA and PVA—that is why authors proposed to add the dopamine into the electrospinning solution -Crosslinked mats degrade more slowly in PBS than in water, with degradation significantly decreasing as crosslinking time increases.	[143]
				Q = 0.4 mL/h				
				L = 12 cm				
SA, PVA, agarose	water	-	ciproflaxin	U = 30 kV		drug release; biomedical application	-The optimal composition of the solution contained 3:7 2.5% (*w*/*v*) SA and 10% (*w*/*v*) PVA, produced smooth, uniform fibers with an average diameter of 185.1 nm. However, thin fibers were formed using a 1:2:1 ratio of 2.5% SA:10% PVA:0.5% agarose (M = 122.5 nm). These SA-PVA-Ag fibers still showed beading and thus were not drug–loaded -The addition of agarose made electrospinning difficult due to the low solubility of agarose in water -Charges on polymers can affect the release of drugs from carriers -Combining alginate with PVA or agarose via electrospinning should capitalize on the properties of both biocompatible materials in each fiber type to create a unique drug release profile for each scaffold	[45]
				Q = 7.5 μL/min				
				L = 10 cm				
SA, PEO	water	Triton X-100	levoflaxin	U = 15 kV	-	biomedical application	-SA-based nanofibers were 235 ± 43 nm in diameter -Nanofibers demonstrated effective antibacterial activity against *Staphylococcus aureus* and *Pseudomonas aeruginosa* -A smaller fiber diameter can be achieved by using a different solvent, such as HAc, which evaporates more rapidly, resulting in thinner fibers -As the size of the nanofibers decreases, their surface area increases	[140]
				Q = 0.4 mL/h				
				L = 15 cm				
SA, PVA, dextran	water		clotrimazole	U = 15–17 kV		drug delivery—healing patches	-Fiber loaded with clotrimazole showed higher antifungal properties -Mats inhibited the growth of *Candida albicans* and *Candida dubliniensis* -Bioadhesion of the electrospun mat was two-fold higher than of the film due to the higher surface area. Lack of plasticizer in the formulation of nanofiber makes it more favorable from the perspective of safety and industrial production	[145]
				Q = 0.7 mL/h				
				L = 12 cm				
SA, PVA	water		amoxicillin	U = 25 kV	GA	tissue engineering—wound healing	-Diameter of fibers was 201.7 ± 30.9 nm -Average diameters of the nanofibers increased after GA crosslinking due to the flattening of the nanofibers -The nanofibrous mat has no cytotoxic effect on human normal keratinocyte cells -The lower the GA concentration, the higher the number of fusions at junction points of the nanofibers -Due to its absorption properties, the use of SA is important in the treatment of wounds and the absorption of exudate	[142]
				Q = 0.5 mL/h				
				L = 15 cm				
SA, PEO	water		simvastatin	U = 22 kV (−5 kV)		tissue engineering—wound healing	-It was possible to obtain SA fibers with liposomes containing simvastatin and BHA (antioxidant) –Nanofibers were thicker—more than 300 nm in diameter -Considering poor solubility in water of simvastatin and the aim to avoid using organic solvents, green electrospinning was achieved by incorporating polymers into liposomal dispersions -Nanofibers with liposomes and antioxidants showed faster release of simvastatin (important in wound healing)	[148]
				Q = 600 uL/h				
				L = 15 cm				
				T = 37 °C				
				RH = 15%				
SA, PVA	water	Triton X-100	ciproflaxin	Q = 0.3 mL/h	-	tissue engineering—wound healing	-70% of encapsulated drug was released during the first 10 h; the drug was fully released after 48 h -The drug release rate in a polymer matrix system can be dissolution and diffusion-controlled -Ciproflaxin encapsulated in PVA/SA fibers fully inhibited the growth of *P. aeruginosa* and *S. aureus* -Viproflaxin at the concentrations of 0.5 μg/mL and 1 μg/mL incorporated into PVA/SA fibers demonstrated antimicrobial activity -Addition of SA to the electrospinning solution leads to a smaller fiber diameter -Electrospun fibers had a high water uptake capacity	[144]
				L = 14 cm				
				T = 20–25 °C				
				RH = 35–50%				
SA, PVA	water	-	naftifine	U = 15 kV Q = 1 mL/h L = 15 cm	25% GTA vapor	tissue engineering—wound healing	-Optimum ratio of PVA:SA mixture was 8:2 -Average fiber diameter was 457.71 nm -19% of the drug was released within the first 30 min -Addition of the drug caused an increase in conductivity -Crosslinking process improved the hydrophilicity and mechanical properties of fibers -The solubility of the drug in the polymer solution affects the drug entrapment efficiency in nanofibers	[149] ^1^

^1^ L-needle-to-collector distance; Q-feed rate; RH-humidity; T-temperature; U-voltage.

**Table 5:** Alginate—based fibers with added nanoparticles.

Polymers	Solvents	Surfactant	Nanoparticle	Electrospinning Conditions	Crosslinking	Application	Results/Conclusion/Problems/Perspectives	Reference
SA, PEO	water, DMSO	Triton X-100	Ag NPs (by immersion in silver nitrate)	U = 12 kV	after electrospinning nanofiber membrane was immersed in silver nitrate solution for exchange of silver and sodium ions	surface corrosion analysis of bronze artifacts	-Ag–SA nanofiber membranes can successfully detect bronze patina trace	[153]
				Q = 0.4 mL/h				
				L = 12 cm				
SA, PCL	water, chloroform, methanol	-	ZnO NPs	U = 25 kV L = 15 cm Shell solution: Q = 0.2 mL/h Core solution: Q = 1 mL/h T = 25 °C RH = 30%	-	tissue engineering—wound healing	-The average diameter of nanofibers: 187 ± 51 nm, improved tensile strength -Application of ZnO NPs and essential oils increased antimicrobial activity -The scaffold accelerated the healing time with total wound healing over 14 days in mouse models carrying full-thickness wounds compared to the nanofibrous scaffold without additives -While the drug release profile of the membrane was satisfactory, close to 50% of the bioactive agents were not released in 5 days	[152]
SA, PEO	water	-	ZnO NPs	Q = 1.0 μL/s	CaCl_2_	water treatment—removing residual tetracyclines	-Fabricated nanofibers showed great adsorption capacity and rapid adsorption for tetracyclines -Not only does PEO improve the electrospinability of SA but also ZnO NPs, forming a hydrogen bond with SA	[151]
				U = 21 kV				
				L = 15 cm				
oxidized SA, PEO	water, ethanol	Triton X-100	ZnO NPs	Q = 0.8 mL/h U = 18 kV Collector speed: 120 rpm L = 15 cm T = 25 °C RH = 50%	adipic acid dihydrazide	tissue engineering—wound healing	-Membranes with ZnO NPs addition showed exceptional epithelialization, neovascularization, and anti-inflammatory response compared to membranes without ZnO NPs -The biodegradability of membranes can be adjusted by different concentrations of the crosslinking agent-the fibers became morphologically stable with the concentration increase -Oxidizing SA offers several advantages, such as generating multiple functional aldehyde groups, reducing Mw and viscosity, and enhancing biodegradability	[150]
SA, PEO	water	Triton X-100	Ag NPs (by immersion in silver nitrate)	U = 11 kV (without Ag NPs)	BaCl_2_	biomedical and food industrial fields	-Ag NPs slightly increased dimensions and were well distributed in the produced mats -The water–ethanol BaCl_2_ solution was chosen because ethanol acts as a non-solvent for alginate. The 60:40 ratio was optimal, as it allows dissolving of barium salt and not dissolving the alginate	[51]
				U = 15 kV (with Ag NPs)				
				L = 15 cm				
				Q = 0.5 mL/h				
				T = 25 °C				
SA, PEO	water	Triton X-100	ZnO NPs	L = 15 cm	SrCl_3_	without a specified application	-fibers diameter: 70–150 nm -PEO was eliminated by washing fibers in hot ethanol -SA with a low Mw and high M/G ratio or SA with a medium Mw and low M/G ratio should be preferred for combination with ZnO NPs	[65] ^1^
				U = 10–12 kV				
				Q = 0.4–0.75 mL/h				

^1^ L-needle-to-collector distance; Q-feed rate; RH-humidity; T-temperature; U-voltage.

## Data Availability

No new data were created or analyzed in this study. Data sharing is not applicable to this article.

## Abbreviations

The following abbreviations are used in this manuscript:

AAD	Amphiphilic alginate derivative
BWA	Black wolfberry anthocyanin
CPD	Carbon polymer dot
DCM	Dichloromethane
DMF	Dimethylformamide
DMSO	Dimethyl sulfoxide
EDC	1-ethyl-3-(3-dimethylaminopropyl)carbodiimide hydrochloride
FAO	Food and Agriculture Organization
G	Guluronic acid
GA	Glutaraldehyde
GFP	Green fluorescent protein
GRAS	Generally recognized as safe
HAc	Acetic acid
HDF	Human dermal fibroblasts
L	Needle-to-collector distance
LAB	Lactic acid bacteria
M	Mannuronic acid
MRSA	Methicyllin-resistant *Staphylococcus aureus*
MTT	3-(4, 5-dimethylthiazol-2-yl)-2, 5-diphenyltetrazolium bromide
NHS	*N*-Hydroxysuccinimide
NPs	Nanoparticles
O/W	Oil-in-water
OEO	Oregano essential vulgare oil
PAA	Polyacrylic acid
PCL	Polycaprolactone
PDA	Polydopamine
PEO	Polyethylene oxide
PHB	3-hydroxybutanoic acid
PLA	Polylactic acid
PS80	Polysorbate 80
PVA	Polyvinyl alcohol
Q	Feed rate
RH	Humidity
SA	Sodium alginate
SOD	Superoxide dismutase
T	Temperature
TFA	Trifluoroacetic acid
U	Voltage
WHO	World Health Organization
